# Asymmetric Amination of *meso*-Epoxide with Vegetable Powder as a Low-Toxicity Catalyst

**DOI:** 10.3390/molecules25143197

**Published:** 2020-07-13

**Authors:** Yuki Takeuchi, Tatsuhiro Asano, Kazuya Tsuzaki, Koichi Wada, Hiroyuki Kurata

**Affiliations:** 1 Kyowa Pharma Chemical Co., Ltd., Chokeiji, Takaoka, Toyama 933-8511, Japan; tatsuhiro.asano@kyowa-kirin.co.jp (T.A.); kazuya.tsuzaki@kyowa-kirin.co.jp (K.T.); koichi.wada@kyowa-kirin.co.jp (K.W.); 2 Graduate School of Engineering, Fukui University of Technology, Fukui 910-8505, Japan; 3 Organization for Fundamental Education, Fukui University of Technology, Fukui 910-8505, Japan; kurata@fukui-ut.ac.jp

**Keywords:** polysaccharide, asymmetric amination, low-toxicity catalyst, pectin

## Abstract

This paper describes the scope and limitation of substrates subjected to asymmetric amination with epoxides catalyzed by a soluble soybean polysaccharide (Soyafibe S-DN), which we recently discovered from the reaction of 1,2-epoxycyclohexane with cyclopropylamine. Various *meso*-epoxides reacted with various amines afforded the corresponding products with good enantiomeric selectivity. Since it was found that pectin was found to have a catalytic ability after screening commercially available polysaccharides, we studied 33 different vegetable powders having pectic substances, and we found that many vegetable powders showed catalytic ability. These results should guide in using vegetable components as low-toxic catalysts for the production of pharmaceuticals.

## 1. Introduction

**Figure 1:**
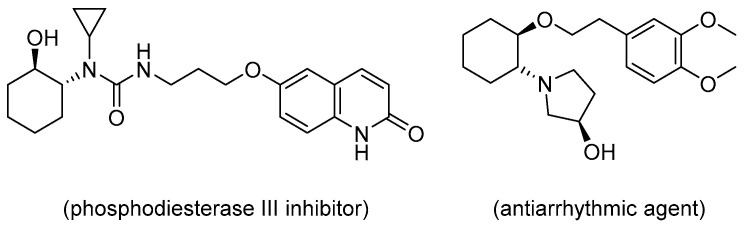
Structures of active pharmaceutical ingredients containing the *trans*-β-aminocyclohexanol moiety.

**Figure 2:**
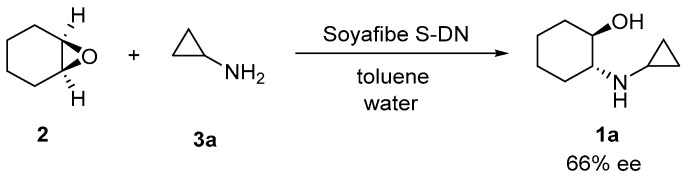
Asymmetric synthesis of (1*R*,2*R*)-2-(cyclopropyl-amino)cyclohexan-1-ol (**1a**) catalyzed by a soy product.

Chiral β-aminoalcohol units are found in many bioactive compounds used for the therapeutic treatment of various diseases [1,2,3]. Chiral *trans*-β-aminocyclohexanol derivatives are particularly useful substrates as key intermediates for the synthesis of 1-cyclopropyl-1-((1*R*,2*R*)-2-hydroxycyclohexyl)-3-(3-((2-oxo-1,2-dihydroquinolin-6-yl)oxy)propyl)urea, which is a phosphodiesterase III inhibitor with vascular hypertrophy effects, and (*R*)-1-((1*R*,2*R*)-2-(3,4-dimethoxyphenethoxy)cyclohexyl)-pyrrolidin-3-ol, which is an antiarrhythmic agent (Figure 1) [4,5,6]. The most effective method to synthesize chiral *trans*-β-aminocycloalkanols is the catalytic asymmetric amination of the corresponding 1,2-epoxycycloalkanes. Although many catalytic systems have been reported for this reaction, most of them are based on organometallic complexes, such as titanium [7,8], scandium [9], cobalt [10], zinc [11], magnesium [12], and niobium [13]. The permissible concentrations of elemental impurity in pharmaceuticals are strictly prescribed by the Q3D guideline established by the International Council for Harmonisation of Technical Requirements for Pharmaceuticals for Human Use. Therefore, a low-toxicity catalyst for pharmaceutical production is highly sought after. In addition, green chemistry protocols must be applied for pharmaceutical production [14]. We have previously reported that soy products, especially the soybean polysaccharide Soyafibe S-DN, are effective catalysts for the asymmetric amination of 1,2-epoxycyclohexane to synthesize (1*R*,2*R*)-2-(cyclopropylamino)cyclohexan-1-ol (**1a**) (Figure 2) [15]. We have also reported that Soyafibe S-DN is reusable without further treatment and has a low environmental impact.

Soyafibe S-DN is composed of soluble soy polysaccharides and is highly safe and easily available [16]. It is made from the cake obtained after soy milk extraction and is used in large quantities as a food additive in the food industry, e.g., as a stabilizer for dairy products [17]. Our results showed that the catalytic activity of Soyafibe S-DN was due to the polysaccharide. In other words, we have developed a new and highly safe catalytic system for the asymmetric amination of epoxides, wherein soybean or the dietary fiber contained in soybean acted as the catalyst. Therefore, it is suitable for use in pharmaceutical manufacturing processes that require the use of highly safe compounds. In this paper, we report the scope of the reactants in asymmetric amination using Soyafibe S-DN. We also report the results of the reaction of 1,2-epoxycyclohexane with cyclopropylamine catalyzed by vegetables other than soy.

## 2. Results and Discussion

### 2.1. Substrate Scope for Epoxide

Initially, to obtain a suitable epoxide, cyclopropylamination was carried out for 136 h on five types of epoxides (**2**, **4**–**7**). The conversion and enantiomeric excess (ee) of the five corresponding *trans*-β-cyclopropylaminoalkanols (**1a**, **4a**–**7a**) obtained are listed in Table 1. Thus, Soyafibe S-DN catalyzed the cyclopropylamination of all the five epoxides studied. In addition, epoxides with 5- or 6-membered ring skeletons were found to be particularly suitable. In terms of enantioselectivity, 1,2-epoxycyclopentane and 1,2-epoxycyclohexane were the desirable substrates. The absolute configurations of the products were not identified except for **1a** (**1a** has (*R*,*R*) configuration). The reaction time for studying the epoxide scope was 136 h, but it is presumed that the reaction proceeded faster for 1,2-epoxycyclopentane and 1,2-epoxycyclohexane. Indeed, in the experiment using 1,2-epoxycyclohexane at a 35 g scale, the conversion to **1a** was 95% in 26 h. This section may be divided by subheadings. It should provide a concise and precise description of the experimental results, their interpretation, as well as the experimental conclusions that can be drawn.

### 2.2. Substrate Scope for Amine

The amine substrate scope was studied using **2** as the substrate. The conversion and ee of the corresponding trans-β-aminocyclohexanols are listed in Table 2. The absolute configurations of the products were not identified except for **1a** (**1a** has (*R*,*R*) configuration). Table 2 shows that the Soyafibe S-DN-catalyzed amination of epoxides proceeded with a wide range of amines, including ammonia. Aliphatic amines are particularly suitable but are unsuitable when they bear bulky substituents such as the *tert*-butyl group. The amines that afforded relatively high ee in this reaction had moderately hindered substituents, such as cyclopropyl, isopropyl, propargyl, 3-pentyl, and 2-phenylethyl groups. Except for amines bearing branched-chain substituents such as propargyl and 2-phenylethyl, the aforesaid substituents and nucleophiles bearing these substituents are suitable for this catalytic system. Similarly, since cyclopentylamine has a branched structure similar to the cyclopentane ring, the ee of the product was moderate.

### 2.3. Proposed Reaction Mechanism

**Figure 3:**
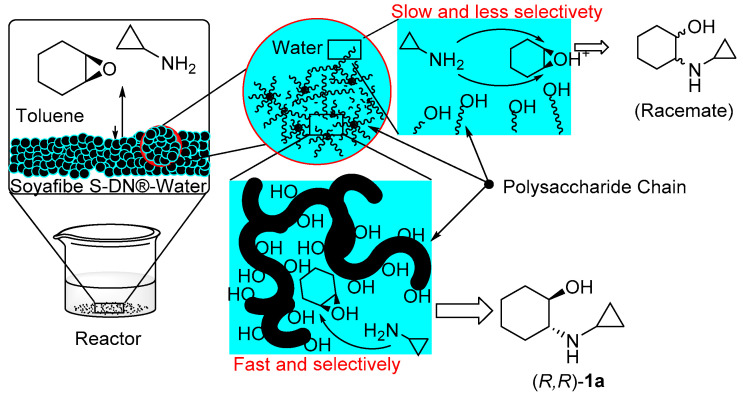
Proposed reaction scheme.

Based on the obtained results, we propose the reaction scheme as shown in Figure 3**.** The structures of the soluble soy polysaccharides are composed of rhamnogalacturonan, galacturonan, homoarabinan, homogalactan, xylan, and neutral sugars [17,18]. The addition of water is indispensable for the reaction in the presence of Soyafibe S-DN; the amount of water suitable for this reaction was found to be 0.2–0.5 times the weight of Soyafibe S-DN. Any deviation from this amount of water resulted in a decreased conversion rate and ee [15]. An aqueous solution of a small amount of Soyafibe S-DN covers the grain. Therefore, Soyafibe S-DN can act as an emulsifier that can be transferred to water from toluene.

The amination of aliphatic epoxides with aliphatic amines in water in the absence of any catalyst afforded the corresponding β-aminoalcohols in good yields [19]. We believe that the reaction is promoted by the water-containing polysaccharide chain by fixing the epoxide and drawing the amine in its vicinity. Epoxide amination was slow in the solution around the grain, while the epoxide entered between the polysaccharide chains in the grain relatively fast. The active site of this catalyst is the undissolved polysaccharide moiety in the solution, similar to a sponge soaked in water. For large epoxides such as **6**, it is difficult to reach the active site, and the ring-opening reaction with amines proceeds slowly. On the other hand, epoxide **7**, which has a smaller backbone than the other epoxides, is difficult to immobilize on the chain despite its capability to enter the active site. Therefore, the conversion is moderate.

The enantioselectivity of the product is attributable to the structure of the polysaccharide catalyst. When the reaction was conducted using a highly nucleophilic amine, the reaction progressed more rapidly in the solution of polysaccharide; thus, a lower ee was obtained (Table 2, entries 9, 11, and 12). When the product was obtained with a high ee, it was presumed that the reaction proceeded in the network of the sugar chain constituting the undissolved Soyafibe S-DN. The epoxide is fixed by the network and is attacked by the amine from a less sterically hindered side. In the vicinity of this site, there is space where the amine can be fixed to some extent, and an amine that can fit here gives the corresponding product in high ee (Table 2, entries 1, 3, 5, 7, and 10). By contrast, it is estimated that amines with structures that are difficult to fit in this site yield products in lower ee.

### 2.4. Screening of Polysaccharides as Catalysts

Soyafibe S-DN is reportedly composed of pectin-like polysaccharides [18], which are responsible for the activity of this catalyst in the asymmetric amination of epoxides. Therefore, we investigated whether commercially available polysaccharides can catalyze the cyclopropylamination of **2**. The reaction was carried out for 16 h. The conversion and the ee values of the obtained product **1a** for different polysaccharide catalysts are shown in Table 3.

Pectin, consisting of galacturonan and rhamnogalacturonan, functioned as a catalyst to give **1a**. On the other hand, pectic acid obtained by hydrolysis of the methyl ester of pectin afforded no products. When 0.6 mmol of **3a** was used as the substrate, catalysis by neither pectin nor pectic acid gave any products; **3a** was also not detected by GC in the reaction solution. Since the amine was retained on the carboxyl group of pectin and pectic acid, and no reaction occurred, the amount of **3a** was increased to 1.2 mmol. Consequently, the amine was detected in both the experiments, but the product was obtained only with pectin. The following polysaccharides did not function as catalysts: dextran composed only of glucose, chitosan composed of glucosamine and its acetate, carrageenan composed of sulfated galactose, curdlan composed of 1,3-glucan, arabinogalactan consisting of arabinose and galactose, gum arabic composed of arabinogalactan, and xanthan gum composed of glucose, mannose, and glucuronic acid. The components common to pectin and Soyafibe S-DN are galacturonan and rhamnogalacturonan [17,18]. Therefore, they could play important roles in the catalysis of asymmetric amination of epoxides. The difference in activity between pectin and Soyafibe S-DN can be attributed to the different shapes of the molecules. Soyafibe S-DN is highly branched star-shaped, while pectin has few branches and is rod-shaped [18]. Since the catalyst was not dissolved in the reaction system, the catalytic activity depended on the surface area. Hence, we assume that the catalytic activity is higher for Soyafibe S-DN.

### 2.5. Screening of Vegetables

Pectic substances are found in many plant species [20]. Therefore, we next screened the edible parts and seeds of plants other than soybean for catalysis of the reaction of **2** with **3a**. The reaction was carried out for 16 h.

The 33 kinds of plants screened herein were obtained from the market as food. The commercially available vegetable powders (carrot, pumpkin, lotus root, turmeric, tomato, coffee, citron, tea, garlic, nutmeg, basil, ginger, rice barn, and wheat germ) were used without further processing. Vegetables not commercially available in powdered form (peanut, almond, kiwifruit, apple, pomelo seed, pistachio, kidney bean, green pea, banana, chickpeas, olive, ginkgo, red bean, pepper, perilla, sunflower seed, kelp, seaweed, and hijiki) were dried under reduced pressure and then pulverized and washed with hexane to remove oil, and the washed and dried powders were used as catalysts. The results for reactions with conversion >3% are listed in Table 4. Many kinds of plants can promote this reaction. The conversion and ee values of **1a** differed depending on the type of plant used to prepare the catalyst, due to the differences in structure and polysaccharide content of the vegetables. The use of kiwifruit, carrot, pomelo, and pumpkin powders as catalysts gave **1a** with >50% ee. When tea powder was used as the catalyst, the highest conversion (29%) among the plants used was achieved; however, the ee achieved was as low as 28%. The use of turmeric powder as the catalyst gave the (*S*,*S*) isomer-rich product with 14% ee.

### 2.6. Evaluation of Catalytic Performance of Carrot Powder

Carrot powder was identified as the most effective and readily available among the screened plants. Therefore, the amination of **2** with five different C-3 amines were carried out for 136 h using carrot powder as the catalyst to examine the compatibility of the substrate. The results are summarized in Table 5.

When **2** was reacted with amines having branched substituents, **1a** and **1c** were obtained with moderate ee. On the other hand, when amines with linear substituents were used, **1b**, **1d**, and **1e** were obtained with low ee. The conversion and the ee of all the products obtained using carrot powder as the catalyst were lower than those obtained using Soyafibe S-DN. The difference in these results could be due to the different preparation methods of carrot powder and Soyafibe S-DN. Soyafibe S-DN is produced by extracting polysaccharides from the cake obtained after soy milk extraction and contains a large amount of pectin-like substances. On the other hand, carrot powder is prepared by drying the root; therefore, it contains many ingredients other than the polysaccharides, and the pectin content is lower than that in Soyafibe S-DN. Since pectin and pectin-like substances are the catalytically active ingredients, catalysts with higher pectin contents have higher activities. Pectin and pectin-like substances are some of the most complex natural polysaccharides [21,22], and the correlation between structure and catalytic activity could not be clarified. However, if the correlation between the structure of pectin and substrate is determined, a new catalytic system using a polysaccharide can be pioneered.

## 3. Conclusions

In summary, we investigated the suitability of using a soluble soybean polysaccharide (Soyafibe S-DN) as a catalyst for the asymmetric amination of epoxides. Soyafibe S-DN catalyzed the amination of epoxides with 5-membered or 6-membered ring backbones; particularly, the cyclopropylamination of 1,2-epoxycyclohexane and 1,2-epoxycyclopentane gave the corresponding *trans*-β-cyclopropylaminoalcohols with good enantioselectivities. Many of the amines that reacted with 1,2-epoxycyclohexane in the presence of this catalyst system to give the corresponding optically active *trans*-β-aminocyclohexanols. The ee of the product was relatively high when an amine with a branched structure was used as the substrate; particularly, 3-aminopentane showed the highest ee (81%) in this study. We have shown that the asymmetric amination of 1,2-epoxycyclohexane proceeded even when using the powder of different types of vegetables containing pectin and pectin-like substances as catalysts other than Soyafibe S-DN. These results suggested that the pectin or pectin-like substances in the catalyst play important roles in catalytic function. The catalytic activity can be enhanced by purifying the pectin contained in various vegetables. Therefore, we have pioneered a new research topic to identify polysaccharides contained in vegetables as low-toxicity catalysts with low environmental impact for pharmaceutical production.

## 4. Materials and Methods

### 4.1. General Procedure

Conversion and enantiomeric excess (ee) were determined by GC analysis on a SHIMADZU GC-2010 or by HPLC analysis on an Agilent 1100 Series instrument by comparison with synthesized racemic compounds. NMR spectra were recorded on a JEOL AL-300 (^1^H NMR 300.40 MHz, ^13^C NMR 75.45 MHz) spectrometer. ^1^H NMR chemical shifts were reported in parts per million (ppm) relative to tetramethylsilane (0 ppm) in CDCl_3_, except for the broad peaks. ^13^C NMR chemical shifts for were reported in parts per million (ppm) relative to CDCl_3_ (77.16 ppm) with complete proton decoupling. High-resolution electrospray-ionization mass spectra (HRMS (ESI)) were recorded with on a Waters Xevo G2-XS QTof mass spectrometer.

### 4.2. Materials

All the reagents were obtained commercially and used as received, unless otherwise noted. The solvents, ammonia, dimethylamine, triethylamine, chlorotrimethylsilane, and pectin (from citrus) were purchased from KANTO CHEMICAL Co., Inc. (Tokyo, Japan). Epoxides, amines other than the ones mentioned above, dextran, chitosan, carrageenan, curdlan, (+)-arabinogalactan (from lurch wood), and xanthan gum were purchased from Tokyo Chemical Industry Co., Ltd. (Tokyo, Japan). Gum arabic was purchased from NACALAI TESQUE, Inc. (Kyoto, Japan). Soyafibe S-DN was purchased from Fuji Oil Co., Ltd. (Osaka, Japan). Pumpkin (powdered), carrot (powdered), tomato (powdered), and lotus (powdered) were purchased from Kodama Foods Co., Ltd. (Hiroshima, Japan). Turmeric (powdered) was purchased from NIPPON FUNMATSU YAKUHIN Co., Ltd. (Osaka, Japan). Kiwifruit, pomelo, pistachio, potato (mashed), citron, apple, tea, kidney beans, and green pea were obtained from a general store.

### 4.3. Procedure and Analytical Data: General Procedure for Pretreatment of Vegetable

Approximately 5 g of vegetable pieces were dried under reduced pressure in a desiccator at 60 °C and were powdered using a pulverizer. The obtained powders were washed with hexane and dried under reduced pressure.

### 4.4. General Procedure for Catalytic Amination of Epoxide with Amine at the 0.5 mL Scale

A mixture of epoxide (0.49 mmol), amine (0.60 mmol), and a catalyst (100 mg) in toluene (0.42 mL) and water (0.02 mL) was shaken at 40 °C for the specified time. After removing the catalyst by filtration, the filtrate was analyzed by GC and HPLC. The conversion was calculated as follows, using the concept of effective carbon number (ECN) [23]:

Conversion (%) = 100 × (A_p_/R_p_)/((A_p_/R_p_) + (A_s_/R_S_))
A_p_ = peak area for aminoalcohol from filtrate
A_S_ = peak area for epoxide from filtrate
R_p_ = response of aminoalcohol
R_S_ = response of epoxide

### 4.5. General Procedure for Trimethylsilylation of the Product

The filtered amination solution was concentrated and redissolved in dichloromethane, and then chlorotrimethylsilane and triethylamine were added. The reaction mixture was stirred, and the solid precipitate was removed by filtration. The obtained solution was used for GC-analysis without further purification.

### 4.6. General Procedure for Benzoation of the Product

The filtered amination solution was concentrated and redissolved in acetonitrile, and then benzoyl chloride and triethylamine were added. The reaction mixture was stirred for 30 min, and the precipitated solid was removed by filtration. The obtained solution was used for HPLC analysis without further purification.

### 4.7. General Procedure for Synthesis of Racemic Aminoalcohol

A mixture of epoxide (15 mmol), amine (15 mmol), and LiCl (0.13 g, 3.1 mmol) in methanol (8 mL) was stirred at 50 °C for 48 h. The reaction mixture was concentrated under reduced pressure to obtain a crude oil. The crude oil was purified by bulb-to-bulb distillation.

*trans-2-(Cyclopropylamino)cyclohexan-1-ol* (**1a**): This compound was used for analysis without purification. The ee value and the conversion were determined by GC (Supelco β-DEX 120, 30 m × 0.25 mm × 0.25 μm; carrier gas, He (pressure 94 kPa); column temperature, 120 °C); Retention time(*t*_R_) of (*S*,*S*)-**1a**, 26.9 min; *t*_R_ of (*R*,*R*)-**1a**, 27.4 min; *t*_R_ of 1,2-epoxycyclohexane, 4.2 min. ^1^H NMR (300.40 MHz, CDCl_3_): δ 0.19–0.55 (4H, m), 0.94–1.07 (1H, m), 1.18–1.30 (3H, m), 1.71–1.76 (2H, m), 2.00–2.06 (1H, m), 2.19–2.36 (3H, m), 3.06–3.14 (1H, m). ^13^C NMR (75.45 MHz, CDCl_3_): δ 5.69 (CH_2_), 7.22 (CH_2_), 24.19 (CH_2_), 24.85 (CH_2_), 27.50 (CH), 30.71 (CH_2_), 33.26 (CH_2_), 63.54 (CH), 72.94 (CH). HRMS (ESI): *m/z* calculated for C_9_H_18_NO^+^ [M+H]^+^: 156.1388, found: 156.1386.

*trans-2-(Cyclopropylamino)cyclopentan-1-ol* (**4a**): bp 105−110 °C (oven temperature)/0.1 mmHg. The conversion was determined by GC (Supelco β-DEX 120, 30 m × 0.25 mm × 0.25 μm; carrier gas, He (pressure 94 kPa); column temperature, 130 °C); *t*_R_ of **4a**, 20.1 and 20.3 min; *t*_R_ of 1,2-epoxycyclopentane, 3.2 min. The ee value was determined by GC (Supelco β-DEX 120, 30 m × 0.25 mm × 0.25 μm; carrier gas, He (pressure 94 kPa); column temperature, 90 °C, (5min) → 130 °C, 20 °C/min) after trimethylsilylation; *t*_R_ of both enantiomer of trimethylsilylated-**4a**, 15.5 min and 15.7 min, respectively. ^1^H NMR (300.40 MHz, CDCl_3_): δ 0.31–0.52 (4H, m), 1.27–1.40 (1H, m), 1.48–1.79 (3H, m), 1.88–1.99 (1H, m), 2.03–2.29 (2H, m), 2.90–2.97 (1H, m), 3.85 (1H, q, *J* = 6.31 Hz). ^13^C NMR (75.45 MHz, CDCl_3_): δ 5.89 (CH_2_), 6.46 (CH_2_), 20.08 (CH_2_), 29.30 (CH), 30.13 (CH_2_), 32.12 (CH_2_), 66.95 (CH), 72.21 (CH). HRMS (ESI): *m/z* calculated for C_8_H_16_NO^+^ [M+H] ^+^: 142.1232, found: 142.1225.

*trans-4-(Cyclopropylamino)tetrahydrofuran-3-ol* (**5a**): This compound was used for analysis without purification. The conversion was determined by GC (Supelco β-DEX 120, 30 m × 0.25 mm × 0.25 μm; carrier gas, He (pressure 94 kPa); column temperature, 155 °C); t_R_ of **5a**, 20.1 min; t_R_ of 3,4-epoxytetrahydrofuran, 2.6 min. The ee value was determined by HPLC (Daicel CHIRALPAK AS-RH, 150 mm × 0.46 mm; mobile-phase A: water, mobile-phase B: acetonitrile, gradient: 15% B in 15 min, 15–80% B in 20 min; flow rate: 1.0 mL/min; temperature: 30 °C; detection: UV absorbance at 254 nm) after benzoate; t_R_ of both enantiomer of benzoated-**5a**, 26.2 min and 28.2 min, respectively. ^1^H NMR (300.40 MHz, CDCl_3_): δ 0.41–0.53 (4H, m), 2.12–2.19 (1H, m), 3.27–3.31 (1H, m), 3.61–3.68 (2H, m), 3.97 (1H, dd, *J* = 9.61, 5.41 Hz), 4.06 (1H, dd, *J* = 9.01, 5.41 Hz), 4.21 (1H, q, *J* = 2.40 Hz). ^13^C NMR (75.45 MHz, CDCl_3_): δ 6.18 (CH_2_), 6.35 (CH_2_), 28.93 (CH), 66.57 (CH), 71.90 (CH_2_), 73.62 (CH_2_), 75.59 (CH). HRMS (ESI): *m/z* calculated for C_7_H_14_NO_2_^+^ [M+H]^+^: 144.1025, found: 144.1016.

*trans-2-(Cyclopropylamino)cycloheptan-1-ol* (**6a**): This compound was used for analysis without purification. The conversion was determined by GC (Supelco β-DEX 120, 30 m × 0.25 mm × 0.25 μm; carrier gas, He (pressure 94 kPa); column temperature, 145 °C); *t*_R_ of **6a**, 27.2 min; *t*_R_ of 1,2-epoxycyloheptane, 4.8 min. The ee value was determined by HPLC (Daicel CHIRALPAK AS-RH, 150 mm × 0.46 mm; mobile-phase A: water, mobile-phase B: acetonitrile, gradient: 15% B in 15 min, 15–80% B in 20 min; flow rate: 1.0 mL/min; temperature: 30 °C; detection: UV absorbance at 254 nm) after benzoate; *t*_R_ of both enantiomers of benzoated-**6a**, 25.3 min and 28.5 min, respectively. ^1^H NMR (300.40 MHz, CDCl_3_): δ 0.22–0.56 (4H, m), 1.21–1.32 (1H, m), 1.39–1.71 (7H, m), 1.89–1.99 (1H, m), 2.07–2.14 (1H, m), 2.21–2.28 (1H, m), 2.33–2.41 (1H, m), 3.06–3.16 (1H, m). ^13^C NMR (75.45 MHz, CDCl_3_): δ 6.20 (CH_2_), 7.13 (CH_2_), 22.08 (CH_2_), 23.71 (CH_2_), 26.55 (CH_2_), 27.62 (CH), 29.90 (CH_2_), 32.75 (CH_2_), 65.76 (CH), 74.90 (CH). HRMS (ESI): *m/z* calculated for C_10_H_20_NO^+^ [M+H]^+^: 170.1545, found: 170.1536.

*trans-3-(Cycloropropylamino)butan-2-ol* (**7a**): bp 135–140 °C (oven temperature)/0.1 mmHg. The ee value and the conversion were determined by GC (Supelco β-DEX 120, 30 m × 0.25 mm × 0.25 μm; carrier gas, He (pressure 94 kPa); column temperature, 110 °C); *t*_R_ of both enantiomers of **7a**, 11.0 min and 11.3 min, respectively; *t*_R_ of *cis*-2,3-epoxybutane, 2.6 min. ^1^H NMR (300.40 MHz, CDCl_3_): δ 0.22–0.31 (1H, m), 0.36–0.55 (3H, m), 1.12 (3H, d, *J* = 6.31 Hz), 1.31 (3H, d, *J* = 11.7 Hz), 2.19–2.26 (1H, m), 2.40–2.50 (1H, m), 3.21–3.30 (1H, m). ^13^C NMR (75.45 MHz, CDCl_3_): δ 6.18 (CH_2_), 7.08 (CH_2_), 16.46 (CH_3_), 19.25 (CH_3_), 27.81 (CH), 60.21 (CH), 70.21 (CH). HRMS (ESI): *m/z* calculated for C_7_H_17_NO^+^ (M+H)^+^: 130.1232, found: 130.1225.

*trans-2-(Propylamino)cyclohexan-1-ol* (**1b**): bp 115–120 °C (oven temperature)/0.2 mmHg. The ee value and the conversion was determined by GC (Supelco β-DEX 120, 30 m × 0.25 mm × 0.25 μm; carrier gas, He (pressure 94 kPa); column temperature, 130 °C); *t*_R_ of both enantiomer of **1b**, 20.0 min and 20.3 min, respectively; *t*_R_ of 1,2-epoxycyclohexane, 4.2 min. ^1^H NMR (300.40 MHz, CDCl_3_): δ 0.90–0.99 (4H, m), 1.21–1.29 (3H, m), 1.42–1.55 (2H, m), 1.71–1.73 (2H, m), 2.02–2.22 (3H, m), 2.39–2.47 (1H, m), 2.70–2.79 (1H, m), 3.10–3.18 (1H, m). ^13^C NMR (75.45 MHz, CDCl_3_): δ 11.69 (CH_3_), 23.60 (CH_2_), 24.39 (CH_2_), 25.03 (CH_2_), 30.43 (CH_2_), 33.52 (CH_2_), 45.54 (CH_2_), 63.47 (CH), 73.46 (CH). HRMS (ESI): *m/z* calculated for C_9_H_20_NO^+^ [M+H]^+^: 158.1545, found: 158.1539.

*trans-2-(Isopropylamino)cyclohexan-1-ol* (**1c**): bp 135–140 °C (oven temperature)/0.1 mmHg. The ee value and the conversion were determined by GC (Supelco β-DEX 120, 30 m × 0.25 mm × 0.25 μm; carrier gas, He (pressure 94 kPa); column temperature, 120 °C); *t*_R_ of both enantiomers of **1c**, 12.9 min and 13.2 min, respectively; *t*_R_ of 1,2-epoxycyclohexane, 4.2 min. ^1^H NMR (300.40 MHz, CDCl_3_): δ 0.82–0.94 (1H, m), 1.00 (3H, d, *J* = 6.31 Hz), 1.06 (3H, d, *J* = 6.31 Hz), 1.20–1.31 (3H, m), 1.69–1.73 (2H, m), 2.06–2.10 (2H, m), 2.18–2.26 (1H, m), 2.69 (1H, sept, J = 6.31 Hz), 3.09 (1H, m). ^13^C NMR (75.45 MHz, CDCl_3_): δ 22.79 (CH_3_), 24.28 (CH_2_), 24.73 (CH_3_), 25.36 (CH_2_), 31.29 (CH_2_), 32.98 (CH_2_), 45.05 (CH), 60.64 (CH), 73.87 (CH). HRMS (ESI): *m/z* calculated for C_9_H_20_NO^+^ [M+H]^+^: 158.1545, found: 158.1535.

*trans-2-(Allylamino)cyclohexan-1-ol* (**1d**): This compound was used for analysis without purification. The ee value and the conversion were determined by GC (Supelco β-DEX 120, 30 m × 0.25 mm × 0.25 μm; carrier gas, He (pressure 94 kPa); column temperature, 120 °C); *t*_R_ of both enantiomers of **1d**, 34.8 min and 35.2 min, respectively; *t*_R_ of 1,2-epoxycyclohexane, 4.2 min. ^1^H NMR (300.40 MHz, CDCl_3_): δ 0.92–1.04 (1H, m), 1.19–1.31 (3H, m), 1.70–1.72 (2H, m), 1.97–2.07 (2H, m), 2.24–2.32 (1H, m), 3.11–3.26 (2H, m), 3.26–3.42 (1H, m), 5.06–5.22 (1H, m), 5.84–5.97 (1H, m). ^13^C NMR (75.45 MHz, CDCl_3_): δ 24.28 (CH_2_), 24.71 (CH_2_), 30.07 (CH_2_), 33.62 (CH_2_), 49.17 (CH_2_), 62.65 (CH), 73.27 (CH), 115.61 (CH_2_), 126.85 (CH). HRMS (ESI): *m/z* calculated for C_9_H_18_NO^+^ [M+H]^+^: 156.1388, found: 156.1378.

*trans-2-(Propargylamino)cyclohexan-1-ol* (**1e**): This compound was used for analysis without purification. The ee value and the conversion were determined by GC (Supelco β-DEX 120, 30 m × 0.25 mm × 0.25 μm; carrier gas, He (pressure 94 kPa); column temperature, 125 °C); *t*_R_ of both enantiomers of **1e**, 47.1 min and 47.6 min, respectively; *t*_R_ of 1,2-epoxycyclohexane, 4.5 min. ^1^H NMR (300.40 MHz, CDCl_3_): δ 0.92–1.05 (1H, m), 1.21–1.38 (3H, m), 1.68–1.73 (2H, m), 1.96–2.08 (2H, m), 2.24 (1H, t, *J* = 2.40 Hz), 2.40–2.48 (1H, m), 3.21–3.29 (1H, m), 3.47 (2H, dq, *J* = 16.82, 2.40 Hz). ^13^C NMR (75.45 MHz, CDCl_3_): δ 24.31 (CH_2_), 24.56 (CH_2_), 29.79 (CH_2_), 33.80 (CH_2_), 35.35 (CH_2_), 62.01 (CH), 71.30 (C), 73.61 (CH), 82.22 (CH). HRMS (ESI): *m/z* calculated for C_9_H_16_NO^+^ [M+H]^+^: 154.1232, found: 154.1222.

*trans-2-(*tert*-Butylamino)cyclohexan-1-ol* (**1f**): bp 100–105 °C (oven temperature)/0.1 mmHg. The ee value and the conversion were determined by GC (Supelco β-DEX 120, 30 m × 0.25 mm × 0.25 μm; carrier gas, He (pressure 94 kPa); column temperature, 130 °C); *t*_R_ of both enantiomers of **1f**, 15.6 min and 16.0 min, respectively; *t*_R_ of 1,2-epoxycyclohexane, 4.2 min. ^1^H NMR (300.40 MHz, CDCl_3_): δ 0.90–1.19 (1H, m), 1.13 (9H, s), 1.24–1.32 (3H, m), 1.67–1.71 (2H, m), 1.98–2.07 (2H, m), 2.19–2.27 (1H, m), 2.89–2.97 (1H, m). ^13^C NMR (75.45 MHz, CDCl_3_): δ 24.47 (CH_2_), 25.88 (CH_2_), 30.63 (CH_3_), 32.64 (CH_2_), 34.88 (CH_2_), 50,68 (C), 58.13 (CH), 74.31 (CH). HRMS (ESI): *m/z* calculated for C_10_H_22_NO^+^ [M+H]^+^: 172.1701, found: 172.1691.

*trans-2-(Pentan-3-ylamino)cyclohexan-1-ol* (**1g**): bp 150–155 °C (oven temperature)/0.1 mmHg. The ee value and the conversion were determined by GC (Supelco β-DEX 120, 30 m × 0.25 mm × 0.25 μm; carrier gas, He (pressure 94 kPa); column temperature, 130 °C); *t*_R_ of both enantiomers of **1g**, 30.3 min, and 31.3 min, respectively; *t*_R_ of 1,2-epoxycyclohexane, 4.2 min. ^1^H NMR (300.40 MHz, CDCl_3_): δ 0.83–0.94 (7H, m), 1.20–1.56 (7H, m), 1.69–1.73 (2H, m), 2.05–2.09 (2H, m), 2.14–2.22 (1H, m), 2.44–2.52 (1H, m), 3.02–3.10 (1H, m). ^13^C NMR (75.45 MHz, CDCl_3_): δ 8.99 (CH_3_), 10.26 (CH_2_), 24.28 (CH_3_), 25.40 (CH_2_), 26.19 (CH_2_), 26.89 (CH_2_), 31.14 (CH_2_), 32.89 (CH_2_), 56.76 (CH), 61.14 (CH), 74.12 (CH). HRMS (ESI): *m/z* calculated for C_11_H_24_NO^+^ [M+H]^+^: 186.1858, found: 186.1849.

*trans-2-(Cyclopentylamino)cyclohexan-1-ol* (**1h**): bp 170–175 °C (oven temperature)/0.1 mmHg. The ee value and the conversion were determined by GC (Supelco β-DEX 120, 30 m × 0.25 mm × 0.25 μm; carrier gas, He (pressure 94 kPa); column temperature, 155 °C); *t*_R_ of both enantiomers of **1h**, 24.7 min and 25.1 min, respectively; *t*_R_ of 1,2-epoxycyclohexane, 3.4 min. ^1^H NMR (300.40 MHz, CDCl_3_): δ 0.83–0.95 (1H, m), 1.20–1.38 (5H, m), 1.50–1.87 (8H, m), 2.01–2.22 (3H, m), 3.01–3.09 (1H, m), 3.19–3.27 (1H, m). ^13^C NMR (75.45 MHz, CDCl_3_): δ 23.59 (CH_2_), 23.79 (CH_2_), 24.31 (CH_2_), 25.27 (CH_2_), 30.91 (CH_2_), 33.08 (CH_2_), 32.12 (CH_2_), 34.59 (CH_2_), 56.14 (CH), 61.86 (CH), 73.75 (CH). HRMS (ESI): *m/z* calculated for C_11_H_22_NO^+^ [M+H]^+^: 184.1701, found: 186.1693.

*trans-2-(Benzylamino)cyclohexan-1-ol* (**1i**): bp 175–180 °C (oven temperature)/0.1 mmHg. The conversion was determined by GC (Supelco β-DEX 120, 30 m × 0.25 mm × 0.25 μm; carrier gas, He (pressure 94 kPa); column temperature, 185 °C, (35min) → 210 °C, (10 °C/min) → 210 °C, (12 min); *t*_R_ of **1i**, 43.9 min; *t*_R_ of 1,2-epoxycyloheptane, 3.2 min. The ee value was determined by HPLC (Daicel CHIRALCEL OB-H, 250 mm × 0.46 mm; mobile phase, *n*-hexane/2-propanol/diethylamine (980:20:1); flow rate; 0.5 mL/min, column temperature; 40 °C); *t*_R_ of both enantiomers of **1i**, 21.0 min and 28.6 min, respectively. ^1^H NMR (300.40 MHz, CDCl_3_): δ 0.91–1.05 (1H, m), 1.16–1.25 (3H, m), 1.71–1.74 (2H, m), 2.01–2.07 (1H, m), 2.15–2.21 (1H, m), 2.25–2.33 (1H, m), 3.16–3.24 (3H, m), 3.69 (1H, d, *J* = 12.92 Hz), 3.96 (1H, d, *J* = 12.92 Hz), 7.22–7.36 (5H, m). ^13^C NMR (75.45 MHz, CDCl_3_): δ 24.25 (CH_2_), 24.85 (CH_2_), 30.20 (CH_2_), 33.40 (CH_2_), 50.68 (CH_2_), 62.89 (CH), 73.41 (CH), 126.81 (CH), 127.96 (CH), 128.25 (CH), 140.32 (C). HRMS (ESI): *m/z* calculated for C_13_H_20_NO^+^ [M+H]^+^: 206.1545, found: 206.1537.

*trans-2-(2-Phenylethylamino)cyclohexan-1-ol* (**1j**): This compound was used for analysis without purification. The conversion was determined by GC (Supelco β-DEX 120, 30 m × 0.25 mm × 0.25 μm; carrier gas, He (pressure 94 kPa); column temperature, 185 °C, (35min) → 210 °C, (10 °C/min) → 210 °C, (12 min); *t*_R_ of **1j**, 47.4 min; *t*_R_ of 1,2-epoxycyloheptane, 3.2 min. The ee value was determined by HPLC (Daicel CHIRALCEL OB-H, 250 mm × 0.46 mm; mobile phase, *n*-hexane/2-propanol/diethylamine (980:20:1); flow rate: 0.5 mL/min; column temperature: 40 °C; detection: UV absorbance at 254 nm); *t*_R_ of both enantiomers of **1j**, 24.2 min and 32.2 min, respectively. ^1^H NMR (300.40 MHz, CDCl_3_): δ 0.84–0.96 (1H, m), 1.18–1.32 (3H, m), 1.67–1.71 (2H, m), 1.99–2.07 (2H, m), 2.16–2.25 (1H, m), 2.70–2.86 (3H, m), 3.00–3.15 (2H, m), 7.18–7.32 (5H, m). ^13^C NMR (75.45 MHz, CDCl_3_): δ 24.29 (CH_2_), 25.15 (CH_2_), 30.55 (CH_2_), 33.25 (CH_2_), 37.01 (CH_2_), 47.89 (CH_2_), 63.55 (CH), 73.62 (CH), 126.06 (CH), 128.36 (CH), 128.63 (CH), 140.06 (C). HRMS (ESI): *m/z* calculated for C_14_H_22_NO^+^ [M+H]^+^: 220.1701, found: 220.1694.

*trans-2-(Dimethylamino)cyclohexan-1-ol* (**1k**): bp 70–75 °C (oven temperature)/0.2 mmHg. The ee value and the conversion was determined by GC (Supelco β-DEX 120, 30 m × 0.25 mm × 0.25 μm; carrier gas, He (pressure 94 kPa); column temperature, 95 °C); *t*_R_ of both enantiomers of **1k**, 46.5 min and 47.1 min respectively; *t*_R_ of 1,2-epoxycyclohexane, 8.4 min. ^1^H NMR (300.40 MHz, CDCl_3_): δ 1.03–1.31 (4H, m), 1.69–1.78 (3H, m), 2.07–2.33 (8H, m), 3.27–3.35 (1H, m). ^13^C NMR (75.45 MHz, CDCl_3_): δ 20.16 (CH_2_), 23.97 (CH_2_), 25.14 (CH_2_), 33.05 (CH_2_), 39.98 (CH_3_), 69.11 (CH), 69.36 (CH). HRMS (ESI): *m/z* calculated for C_8_H_8_NO^+^ [M+H]^+^: 144.1388, found: 144.1382.

*trans-(2-Hydroxycyclohexyl)pyrrolidine* (**1m**): bp 100–105 °C (oven temperature)/0.1 mmHg. The conversion was determined by GC (Supelco β-DEX 120, 30 m × 0.25 mm × 0.25 μm; carrier gas, He (pressure 94 kPa); column temperature, 130 °C); *t*_R_ of **1m**, 35.4 min; *t*_R_ of 1,2-epoxycyclohexane, 3.4 min. The ee value was determined by GC (Supelco β-DEX 120, 30 m × 0.25 mm × 0.25 μm; carrier gas, He (pressure 94 kPa); column temperature, 130 °C), (30min) → 170 °C, 4 °C/min) after trimethylsilylation; *t*_R_ of both enantiomers of trimethylsilylated-**1m**, 23.0 min and 23.2 min, respectively. ^1^H NMR (300.40 MHz, CDCl_3_): δ 1.17–1.27 (4H, m), 1.69–1.78 (7H, m), 2.05–2.15 (1H, m), 2.42–2.59 (3H, m), 2.64–2.71 (2H, m), 3.29–3.37 (1H, m). ^13^C NMR (75.45 MHz, CDCl_3_): δ 20.99 (CH_2_), 23.43 (CH_2_), 24.03 (CH_2_), 25.16 (CH_2_), 33.13 (CH_2_), 47.03 (CH_2_), 64.78 (CH_2_), 70.51 (CH). HRMS (ESI): *m/z* calculated for C_10_H_20_NO^+^ [M+H]^+^: 170.1545, found: 170.1537.

*trans-2-(Phenylamino)cyclohexan-1-ol* (**1n**): This compound was used for analysis without purification. The conversion was determined by GC (Supelco β-DEX 120, 30 m × 0.25 mm × 0.25 μm; carrier gas, He (pressure 94 kPa); column temperature, 185 °C, (35min) → 210 °C, (10 °C/min) → 210 °C, (12 min); *t*_R_ of **1n**, 44.2 min; *t*_R_ of 1,2-epoxycyloheptane, 3.2 min. The ee value was determined by HPLC (Daicel CHIRALCEL OB-H, 250 mm × 0.46 mm; mobile phase, *n*-hexane/2-propanol/diethylamine (980:20:1); flow rate: 0.5 mL/min; column temperature: 40 °C; detection: UV absorbance at 254 nm); *t*_R_ of both enantiomers of **1n**, 27.1 min and 28.9 min, respectively. ^1^H NMR (300.40 MHz, CDCl_3_): δ 0.98–1.11 (1H, m), 1.24–1.47 (3H, m), 1.70–1.79 (2H, m), 2.10–2.14 (2H, m), 3.10–3.18 (1H, m), 3.31–3.39 (1H, m), 6.70–6.77 (3H, m), 7.15–7.25 (2H, m). ^13^C NMR (75.45 MHz, CDCl_3_): δ 24.15 (CH_2_), 24.82 (CH_2_), 31.42 (CH_2_), 33.09 (CH_2_), 59.89 (CH), 74.25 (CH), 114.17 (CH), 118.07 (CH), 129.18 (CH), 147.73 (C). HRMS (ESI): *m/z* calculated for C_12_H_18_NO^+^ [M+H]^+^: 192.1388, found: 192.1380.

*trans-2-aminocyclohexan-1-ol* (**1o**): This compound was purchased from Sigma-Aldrich Co. Ltd. and used without further purification. The ee value and the conversion was determined by GC (Supelco β-DEX 120, 30 m × 0.25 mm × 0.25 μm; carrier gas, He (pressure 94 kPa); column temperature, 120 °C); *t*_R_ of both enantiomers of **1o**, 18.4 min and 18.8 min, respectively; *t*_R_ of 1,2-epoxycyclohexane, 4.2 min.

## Figures and Tables

**Table 1:**
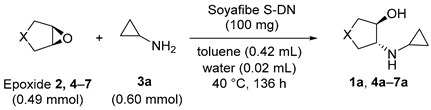
Epoxide scope of the reaction.

Entry	Epoxide	X	Product	Conv. (%)	% ee
Control ^a^	2	-C_2_H_4_-	**1a**	1	-
1	2	-C_2_H_4_-	**1a**	98	65
2 ^b^	2	-C_2_H_4_-	**1a**	95	64
3	4	-CH_2_-	**4a**	98	67
4	5	-O-	**5a**	83	21 ^c^
5	6	-C_3_H_6_-	**6a**	17	35 ^c^
6	7	H, H	**7a**	55	4

Conversions and enantiomeric excess (ee) were determined by GC analysis (date available in Supplementary Materials). ^a^ The reaction was carried out for 22 h without the soluble soybean polysaccharide Soyafibe S-DN. ^b^ Using **2** at 25 g scale for 24 h. ^c^ Determined by HPLC analysis.

**Table 2:**
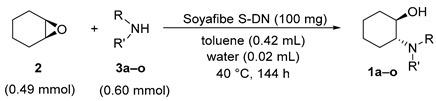
Screening of amines.

Entry	Amine	R	R’	Product	Conv. (%)	% ee
1	3a	Cyclopropyl	H	**1a**	98	65
2	3b	Propyl	H	**1b**	87	39
3	3c	Isopropyl	H	**1c**	97	58
4	3d	Allyl	H	**1d**	96	32
5	3e	Propargyl	H	**1e**	98	53
6	3f	*tert*-Butyl	H	**1f**	4	32
7	3g	3-Pentyl	H	**1g**	37	81
8	3h	Cyclopentyl	H	**1h**	99	48
9	3i	Benzyl	H	**1i**	90	30 ^a^
10	3j	2-Phenylethyl	H	**1j**	100	59 ^a^
11	3k	Methyl	Methyl	**1k**	74	29
12	3m ^b^	–(CH_2_)_4_-		**1m**	99	29
13	3n	Phenyl	H	1n	57	18 ^a^
14	3o	H	H	1o	22	26

Conversions and ee were determined by GC analysis. ^a^ Determined by HPLC analysis. ^b^ **3m** is pyrrolidine.

**Table 3:**
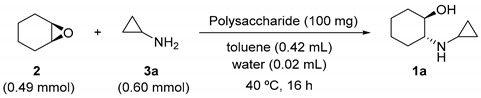
Screening of polysaccharides as catalysts.

Entry	Polysaccharide	Conv. (%) ^a^	%ee ^a^
1	Pectin (from citrus) ^b^	9	42
2	Dextran	1	0
3	Chitosan	2	0
4	Carrageenan	4	0
5	Curdlan	4	−1
6	(+)-Arabinogalactan (from lurch wood)	0	-
7	Gum Arabic	1	0
8	Xanthan gum	0	-
9	Pectinic acid ^b^	0	-

^a^ Conversions and ee were determined by GC analysis. ^b^ 1.2 mmol of cyclopropylamine was used.

**Table 4:**
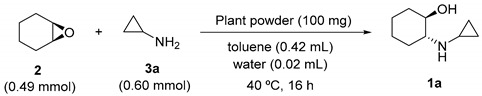
Screening of vegetables.

Entry	Vegetable	Conv. (%) ^c^	% ee ^c^
1	Kiwifruit (peel) ^a^	9	71
2	Carrot ^b^	12	62
3	Pomelo (seed) ^a^	9	54
4	Pumpkin ^b^	8	54
5	Pistachio (seed) ^a^	7	47
6	Potato (Mashed) ^b^	7	33
7	Citron (peel) ^a^	4	32
8	Lotus root ^b^	4	31
9	Apple (seed) ^a^	3	26
10	Tea (leaf) ^a^	29	28
11	Kidney bean (seed) ^a^	5	27
12	Green pea (seed) ^a^	4	14
13	Turmeric ^b^	11	−14

^a^ Commercially available vegetables were used after drying, pulverizing, and degreasing. ^b^ Commercially available dried powders were used without treatment. ^c^ Conversions and ee were determined by GC analysis.

**Table 5:**
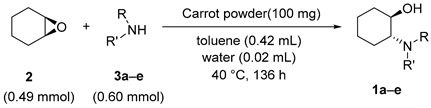
Amination of epoxide using carrot powder.

Entry	Amine	R	R’	Product	Conv. (%)	% ee
1	**3a**	Cyclopropyl	H	**1a**	47	59
2	**3b**	Propyl	H	**1b**	21	13
3	**3c**	Isopropyl	H	**1c**	23	51
4	**3d**	Allyl	H	**1d**	19	8
5	**3e**	Propargyl	H	**1e**	27	2

Conversions and ee were determined by GC analysis.

## Supplementary Materials

GC charts of compounds **1a**, **4a**, **7a**, **1b**, **1c**, **1d**, **1e**, **1f**, **1g**, **1h**, **1k**, **1m** and **1o**. HPLC charts of compounds **5a**, **6a**, **1i**, **1j** and **1n**.
