# Supplementary material for: Asymmetric Amination of meso-Epoxide with Vegetable Powder as a Low-Toxicity Catalyst
Source: Molecules. 2020 Jul 13;25(14):3197. doi: 10.3390/molecules25143197 (PMC7397229; doi:10.3390/molecules25143197)
Supplement: Supplementary file 1 [file molecules-25-03197-s001.pdf]

Supporting Information

Chiral GC chart of **1a**	p. 2
Chiral GC chart of TMS-**4a**	p. 3
Chiral HPLC chart of Bz-**5a**	p. 4
Chiral HPLC chart of Bz-**6a**	p. 5
Chiral GC chart of **7a**	p. 6
Chiral GC chart of **1b**	p. 7
Chiral GC chart of **1c**	p. 8
Chiral GC chart of **1d**	p. 9
Chiral GC chart of **1e**	p. 10
Chiral GC chart of **1f**	p. 11
Chiral GC chart of **1g**	p. 12
Chiral GC chart of **1h**	p. 13
Chiral HPLC chart of **1i**	p. 14
Chiral HPLC chart of **1j**	p. 15
Chiral GC chart of **1k**	p. 16
Chiral GC chart of TMS-**1m**	p. 17
Chiral HPLC chart of **1n**	p. 18
Chiral GC chart of **1o**	p. 19

Chiral GC chart of 1a

Chromatogram

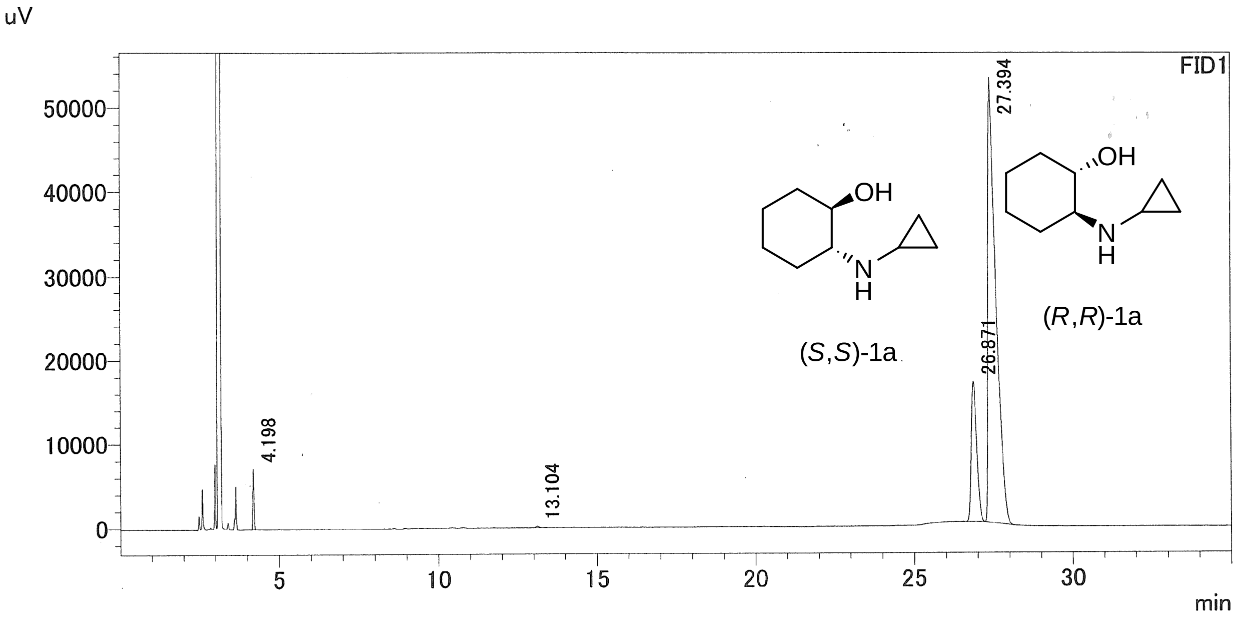

Peak Report

FID1

Peak#	Retention time	Area	Height	Area%	unit	Mark	Name
1	4.198	17943	7136	1.567			
2	13.104	1141	177	0.100			
3	26.871	196730	16745	17.182			
4	27.394	929177	52982	81.151			
Total		1144991	77040				

Chiral GC chart of TMS-4a

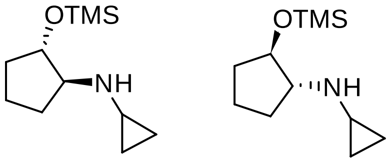

Chromatogram

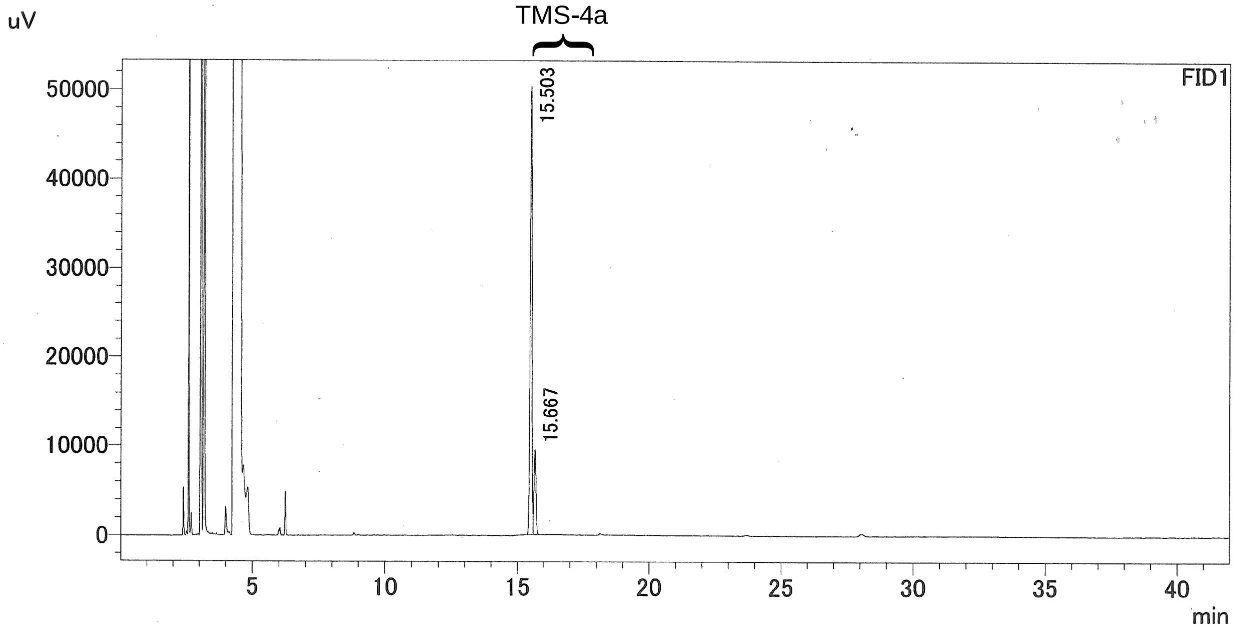

Peak Report

FID1

Peak#	Retention time	Area	Height	Area%	unit	Mark	Name
1	15.503	247538	50308	83.600		V	
2	15.667	48561	9529	16.400		V	
Total		296099	59837				

Chiral HPLC chart of Bz-5a

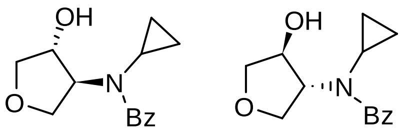

Chromatogram

Bz-5a

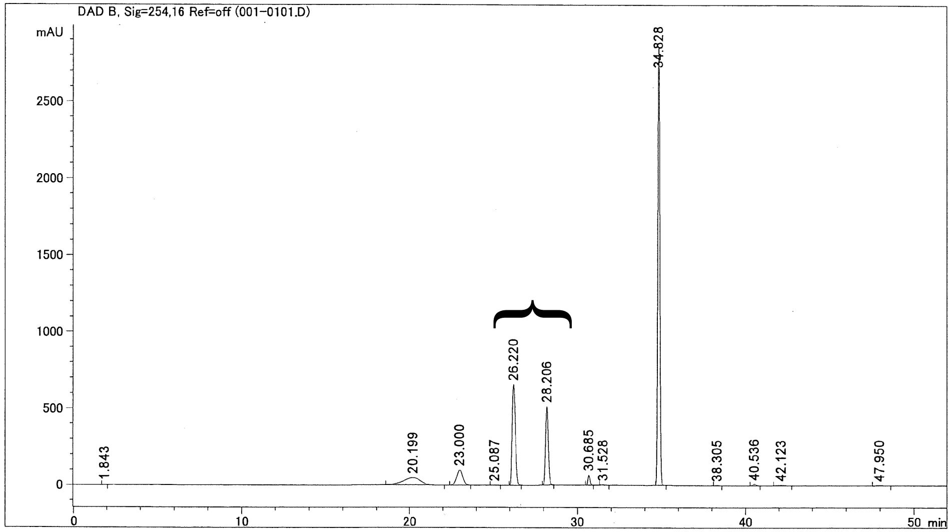

Peak Report

Signal 1: DAD B, Sig=254, 16 Ref=off

Peak #	RT [min]	Type	Width [min]	Area [mAU*s]	Height [mAU]	Area %
1	1.843	BB	0.1321	32.00888	3.16155	0.0714
2	20.199	BB	1.0478	3444.48340	48.07844	7.6857
3	23.000	BB	0.3891	2481.40210	99.44412	5.5368
4	25.087	BB	0.2228	16.79941	1.12106	0.0375
5	26.220	BB	0.2148	8734.53613	658.22095	19.4894
6	28.206	VB	0.1735	5709.16797	514.53467	12.7389
7	30.685	BB	0.1242	528.85016	65.47955	1.1800
8	31.528	BB	0.2143	18.68653	1.34284	0.0417
9	34.828	BB	0.1325	2.35717e4	2850.37769	52.5955
10	38.305	BB	0.1515	12.52412	1.31021	0.0279
11	40.536	BB	0.2018	123.31020	9.47469	0.2751
12	42.123	BB	0.3544	71.43746	2.95094	0.1594
13	47.950	BB	0.4100	71.98547	2.70889	0.1606

1/2

Peak #	RT [min]	Type	Width [min]	Area [mAU*s]	Height [mAU]	Area %
Total :				4.48169e4	4258.20558	

2/2

Chiral HPLC chart of Bz-6a

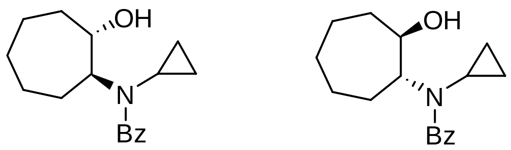

Chromatogram

Bz-6a

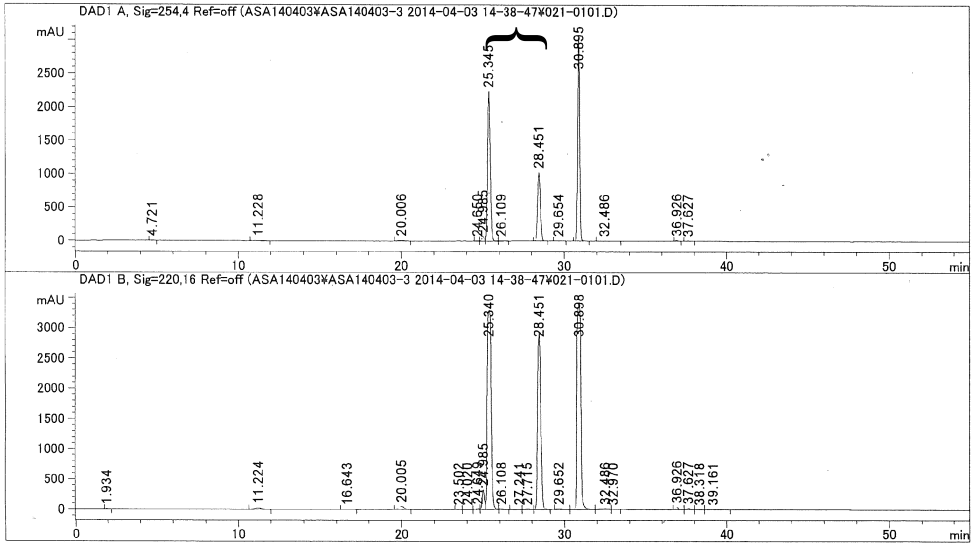

Peak Report

Signal 1: DAD B, Sig=254, 16 Ref=off

Peak #	RT [min]	Type	Width [min]	Area [mAU*s]	Height [mAU]	Area %
1	4.721	VB	0.1351	66.72344	7.40551	0.1029
2	11.228	BB	0.4334	148.25764	5.34860	0.2287
3	20.006	BB	0.3324	246.94748	11.50602	0.3810
4	24.650	BV	0.1593	50.41279	4.93206	0.0778
5	24.985	VV	0.1452	651.16809	69.53995	1.0047
6	25.345	VB	0.1776	2.52227e4	2235.63770	38.9153
7	26.109	BB	0.2200	51.12247	3.27767	0.0789
8	28.451	VB	0.1834	1.20738e4	1025.16479	18.6284
9	29.654	BB	0.2528	50.65626	3.19725	0.0782
10	30.895	BB	0.1323	2.58588e4	3068.27734	39.8968
11	32.486	BB	0.5275	119.51199	2.88351	0.1844
12	36.926	BB	0.1393	168.14557	18.62555	0.2594
13	37.627	BB	0.2180	105.95860	7.72506	0.1635
Total :				6.48142e4	6463.52101	

Signal 1: DAD B, Sig=220, 16 Ref=off

Peak #	RT [min]	Type	Width [min]	Area [mAU*s]	Height [mAU]	Area %
1	1.934	VV	0.1918	91.59538	6.21902	0.0587
2	11.224	BB	0.4442	681.93066	24.09738	0.4373
3	16.643	BB	0.3649	110.49024	4.55629	0.0709
4	20.005	BB	0.3356	1096.35730	50.83878	0.7031
5	23.502	BV	0.1945	56.05373	4.46464	0.0359
6	24.020	VV	0.3375	82.05607	3.17490	0.0526
7	24.649	VV	0.1661	205.14102	18.97254	0.1316
8	24.985	VV	0.1458	3054.40576	324.45630	1.9588
9	25.340	VV	0.2660	5.43255e4	3300.62622	34.8395
10	26.108	VB	0.2157	195.18593	12.96138	0.1252
11	27.241	BV	0.4427	96.05907	2.74193	0.0616
12	27.715	VV	0.2762	188.81850	9.40510	0.1211
13	28.451	VB	0.2173	3.92492e4	2910.47974	25.1709
14	29.652	VV	0.3055	279.87259	13.86436	0.1795
15	30.898	VB	0.2685	5.49418e4	3400.90942	35.2347
16	32.486	BV	0.4511	399.56915	11.79767	0.2562
17	32.970	VB	0.2869	64.87853	3.11555	0.0416
18	36.926	BV	0.1474	373.31137	39.11030	0.2394
19	37.627	VV	0.2245	289.78882	20.55680	0.1858
20	38.318	VV	0.3039	60.26754	2.70518	0.0387
21	39.161	VB	0.3770	88.46028	3.09895	0.0567
Total :				1.55931e5	1.01682e4	

Chiral GC chart of 7a

Chromatogram

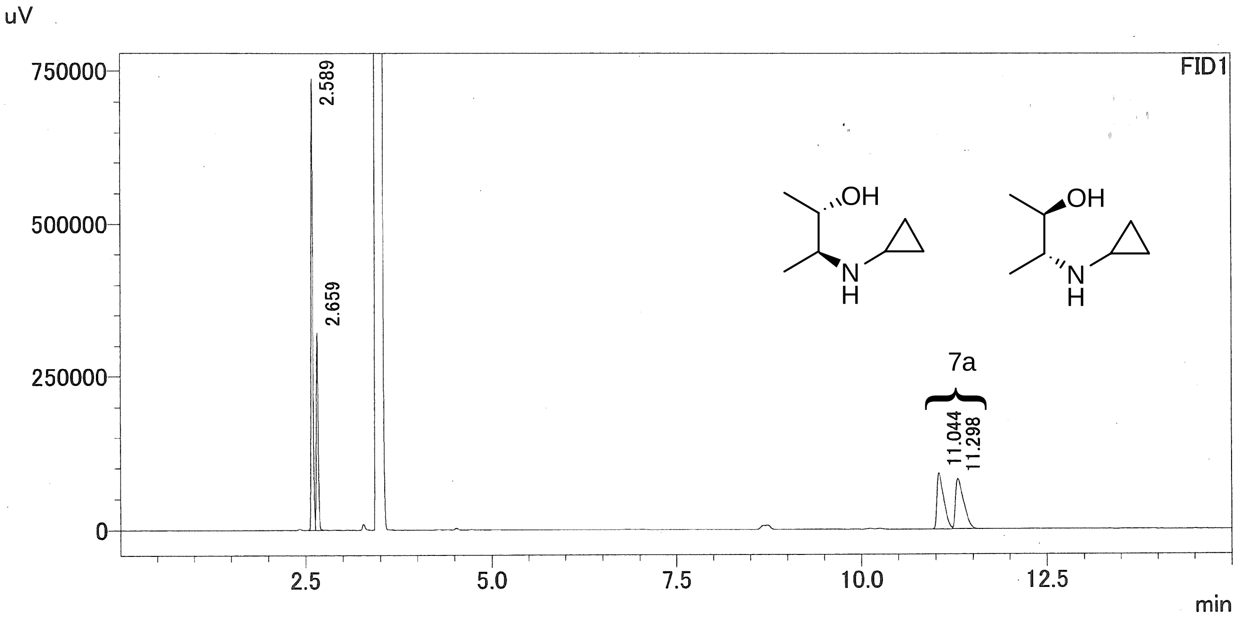

Peak Report

FID1

Peak#	Retention time	Area	Height	Area%	unit	Mark	Name
1	2.589	1251884	726780	42.155		V	
2	2.659	535446	320011	18.030		V	
3	11.044	565014	90431	19.026			
4	11.298	617381	80844	20.789		V	
Total		2969725	1218065				

Chiral GC chart of 1b

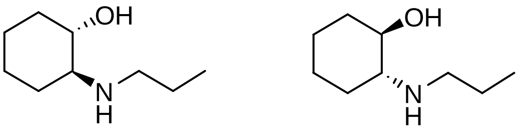

Chromatogram

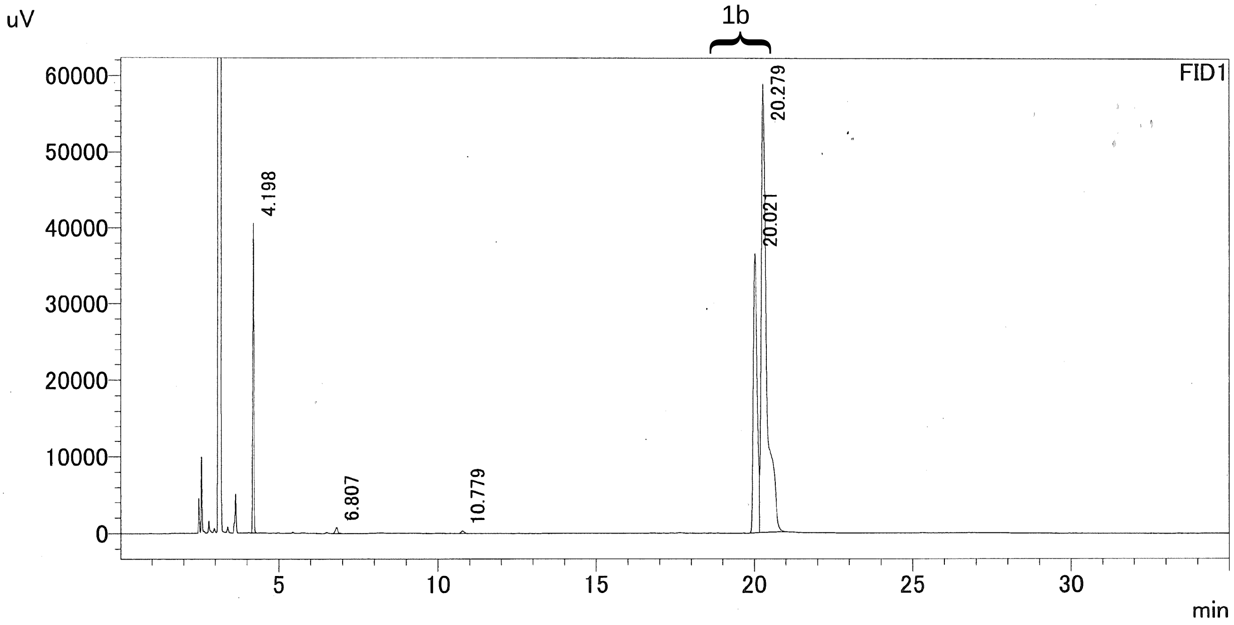

Peak Report

FID1

Peak#	Retention time	Area	Height	Area%	unit	Mark	Name
1	4.198	100552	40381	8.960			
2	6.807	4125	800	0.368			
3	10.779	2025	313	0.180			
4	20.021	310566	36666	27.675			
5	20.279	704938	58902	62.817		V	
Total		1122206	137062				

Chiral GC chart of 1c

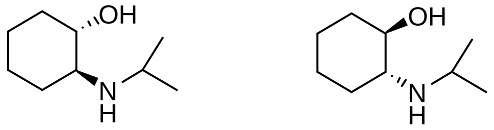

Chromatogram

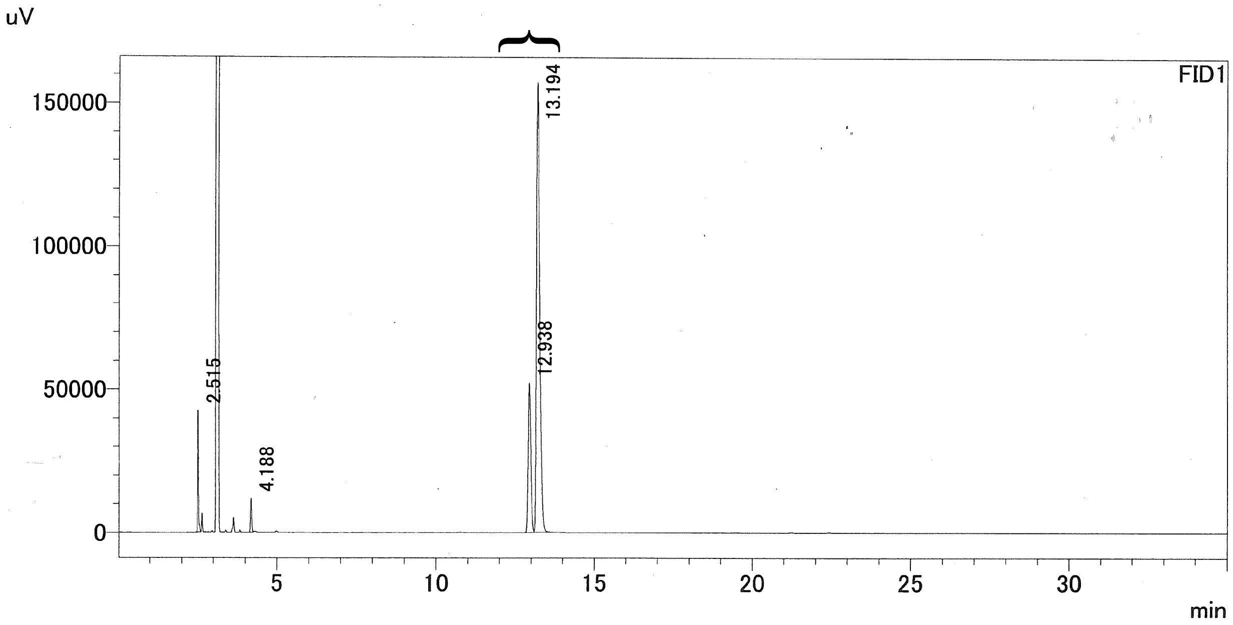

Peak Report

FID1

Peak#	Retention time	Area	Height	Area%	unit	Mark	Name
1	2.515	77446	42400	5.010			
2	4.188	29556	11866	1.912			
3	12.938	299224	52379	19.358			
4	13.194	1139491	157081	73.719		V	
Total		1545717	263726				

Chiral GC chart of 1d

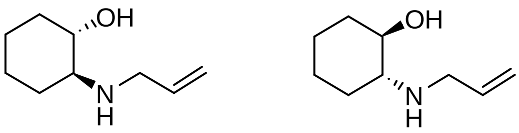

Chromatogram

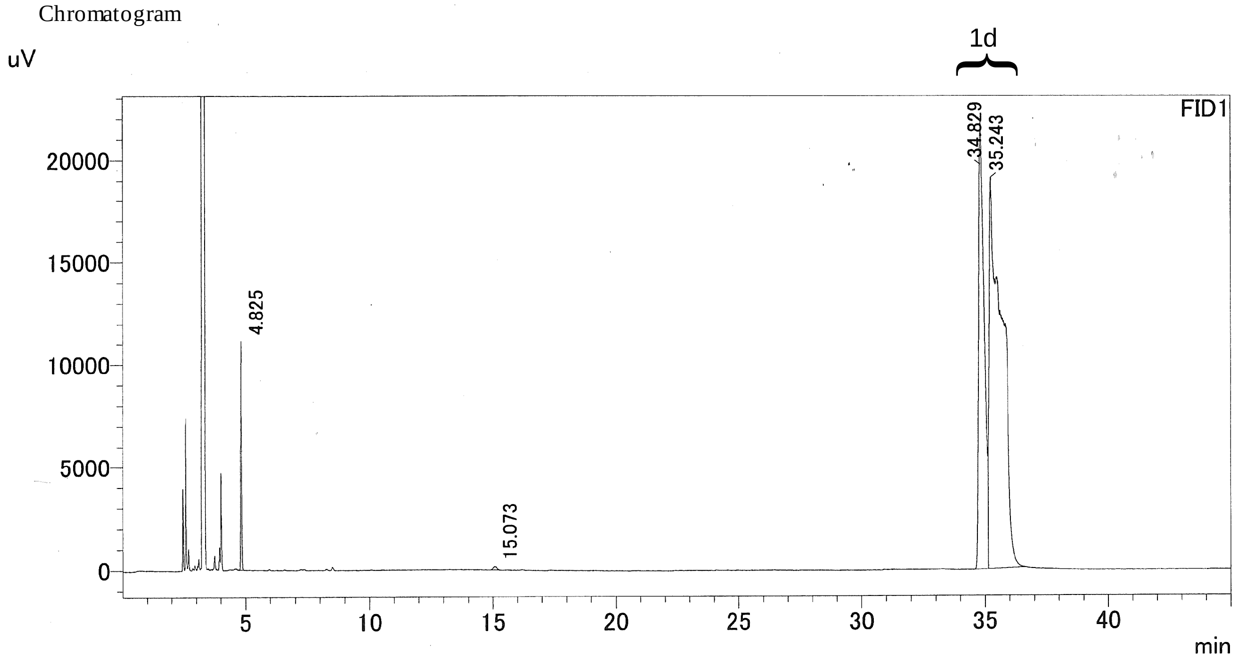

Peak Report

FID1

Peak#	Retention time	Area	Height	Area%	unit	Mark	Name
1	4.825	31353	8707	2.967			
2	15.073	1851	178	0.175			
3	34.829	347428	21764	32.880			
4	35.243	676038	18948	63.978		V	
Total		1056670	49597				

Chiral GC chart of 1e

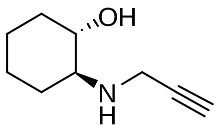

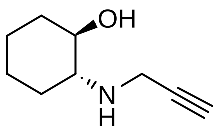

Chromatogram

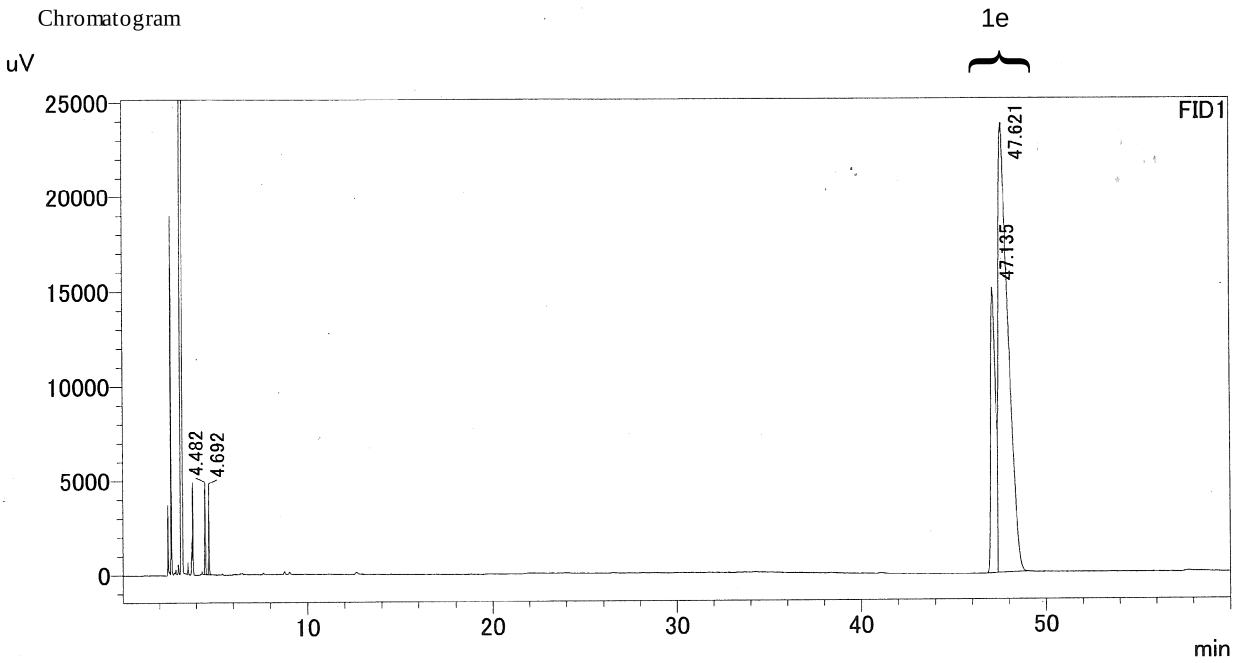

Peak Report

FID1

Peak#	Retention time	Area	Height	Area%	unit	Mark	Name
1	4.482	13075	4324	1.112			
2	4.692	12817	4363	1.090			
3	47.135	272625	15132	23.182			
4	47.621	877501	23707	74.616		V	
Total		1176017	47527				

Chiral GC chart of 1f

Chromatogram

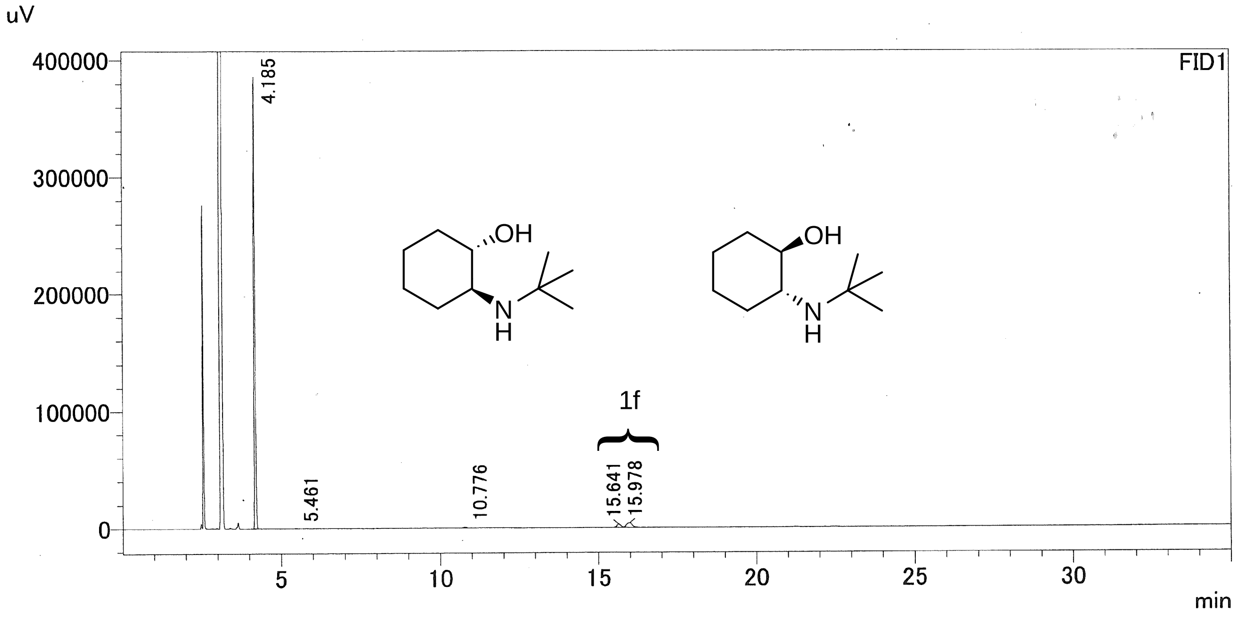

Peak Report

FID1

Peak#	Retention time	Area	Height	Area%	unit	Mark	Name
1	4.185	1008106	383850	93.276			
2	5.461	1089	381	0.101			
3	10.776	5608	847	0.519			
4	15.641	22288	2694	2.062			
5	15.978	43692	3646	4.043		V	
Total		1080783	391418				

Chiral GC chart of 1g

Chromatogram

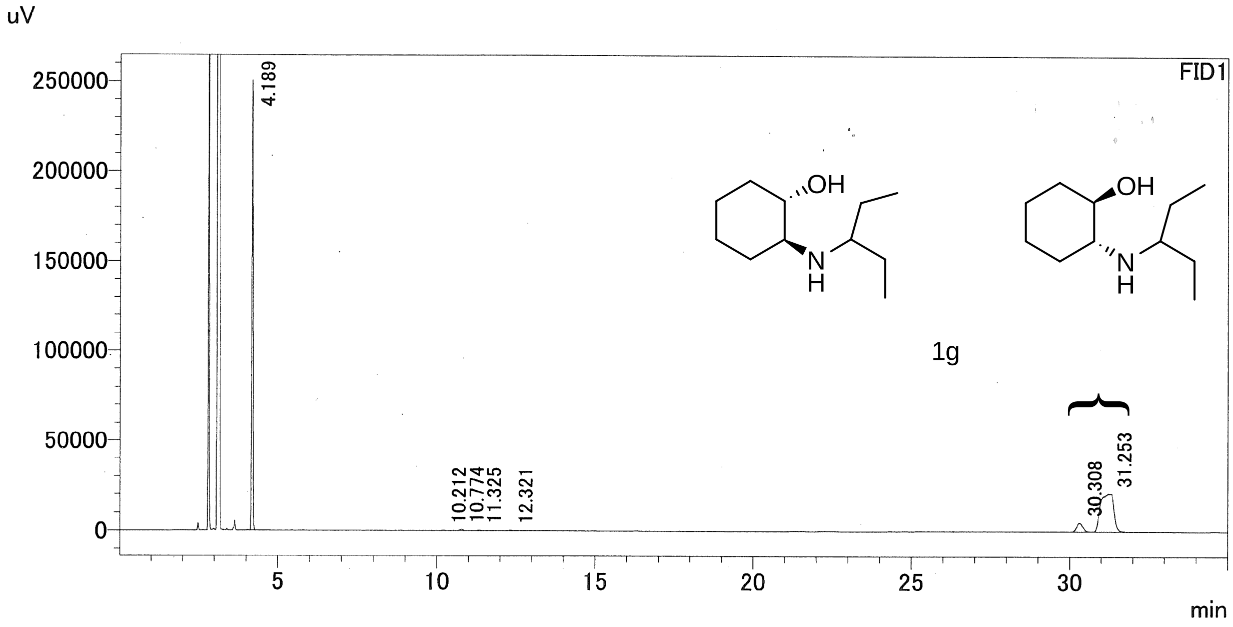

Peak Report

FID1

Peak#	Retention time	Area	Height	Area%	unit	Mark	Name
1	4.189	621492	235011	47.267			
2	10.212	1958	369	0.149			
3	10.774	4852	718	0.369			
4	11.325	1822	336	0.139			
5	12.321	1919	252	0.146			
6	30.308	66328	4895	5.044			
7	31.253	616496	20847	46.887		V	
Total		1314868	262427				

Chiral GC chart of 1h

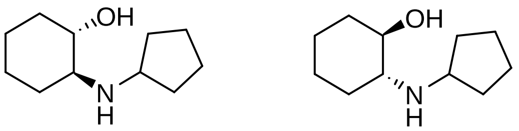

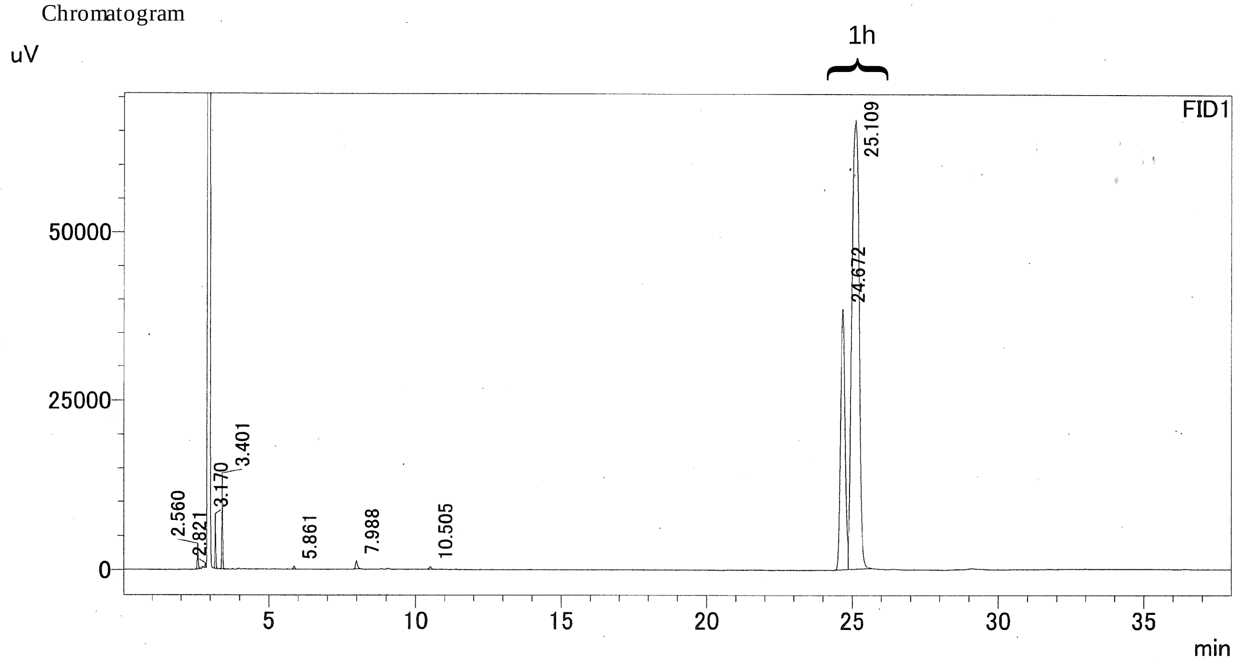

Peak Report

FID1

Peak#	Retention time	Area	Height	Area%	unit	Mark	Name
1	2.560	8281	3821	0.503			
2	2.821	1379	722	0.084		V	
3	3.170	17076	7906	1.037			
4	3.401	30633	13990	1.861		V	
5	5.861	1704	491	0.104			
6	7.988	5798	1206	0.352			
7	10.505	2313	443	0.141			
8	24.672	407183	38741	24.733			
9	25.109	1171975	66746	71.187		V	
Total		1646341	134066				

Chiral HPLC chart of 1i

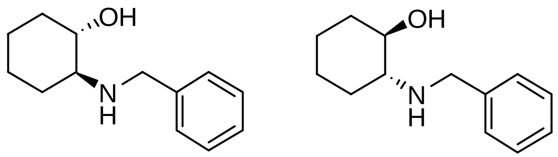

1i

Chromatogram

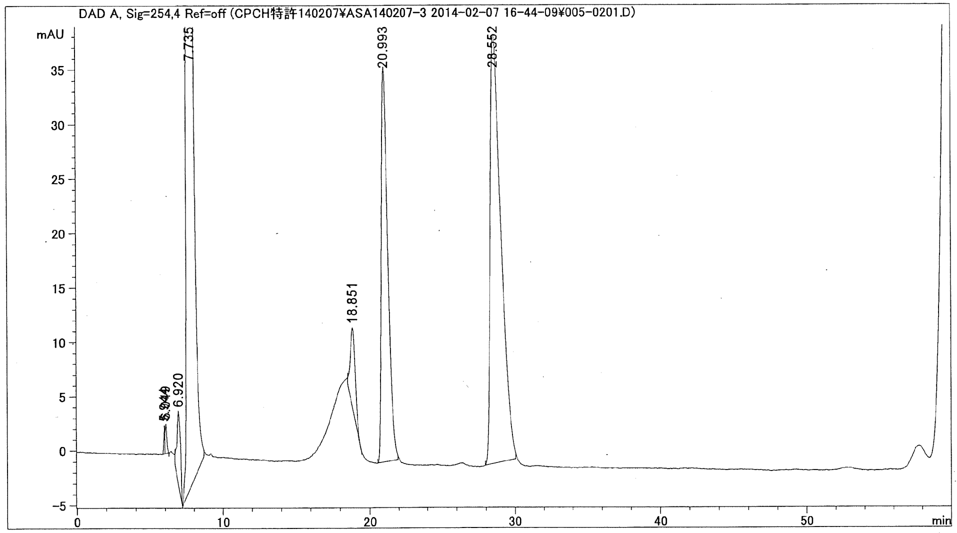

Peak Report

Signal 1: DAD B, Sig=254, 4 Ref=off

Peak #	RT [min]	Type	Width [min]	Area [mAU*s]	Height [mAU]	Area %
1	5.944	BV	0.0807	14.02009	2.60850	0.0482
2	6.049	VB	0.1046	19.31183	2.72727	0.0664
3	6.920	BV	0.2402	112.50687	6.75883	0.3869
4	7.735	VB	0.2019	2.56272e4	2049.35229	88.1235
5	18.851	BB	0.3318	169.21989	7.37249	0.5819
6	20.993	BB	0.4467	1103.02710	36.28101	3.7929
7	28.552	BB	0.7385	2035.73901	39.41897	7.0002

Total : 2.90811e4 2144.51936

==

Chiral HPLC chart of 1j

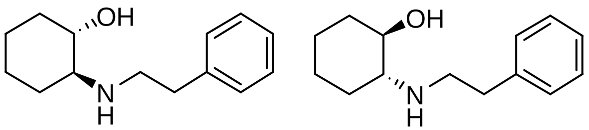

1j

Chromatogram

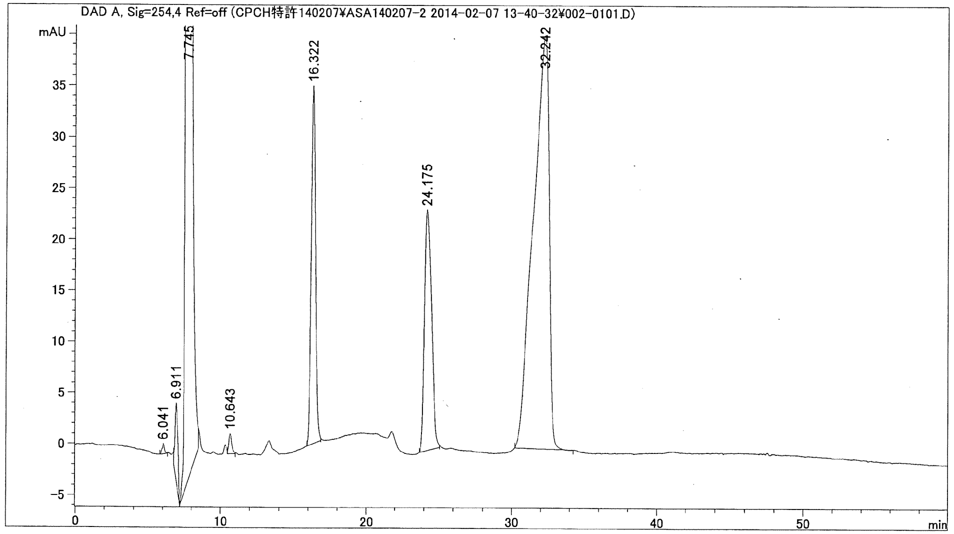

Peak Report

Signal 1: DAD B, Sig=254, 4 Ref=off

Peak #	RT [min]	Type	Width [min]	Area [mAU*s]	Height [mAU]	Area %
1	6.041	BB	0.1540	11.53880	1.01964	0.0366
2	6.911	BV	0.2031	106.09708	7.40927	0.3364
3	7.745	VB	0.2186	2.68276e4	2023.95679	85.0623
4	10.643	VB	0.2158	27.20819	1.96132	0.0863
5	16.322	BB	0.3149	709.83966	34.95560	2.2507
6	24.175	BB	0.5022	787.91797	23.55886	2.4982
7	32.242	BB	0.9993	3068.57324	40.44015	9.7295

Total : 3.15388e4 2133.30163

==

Chiral GC chart of 1k

Chromatogram

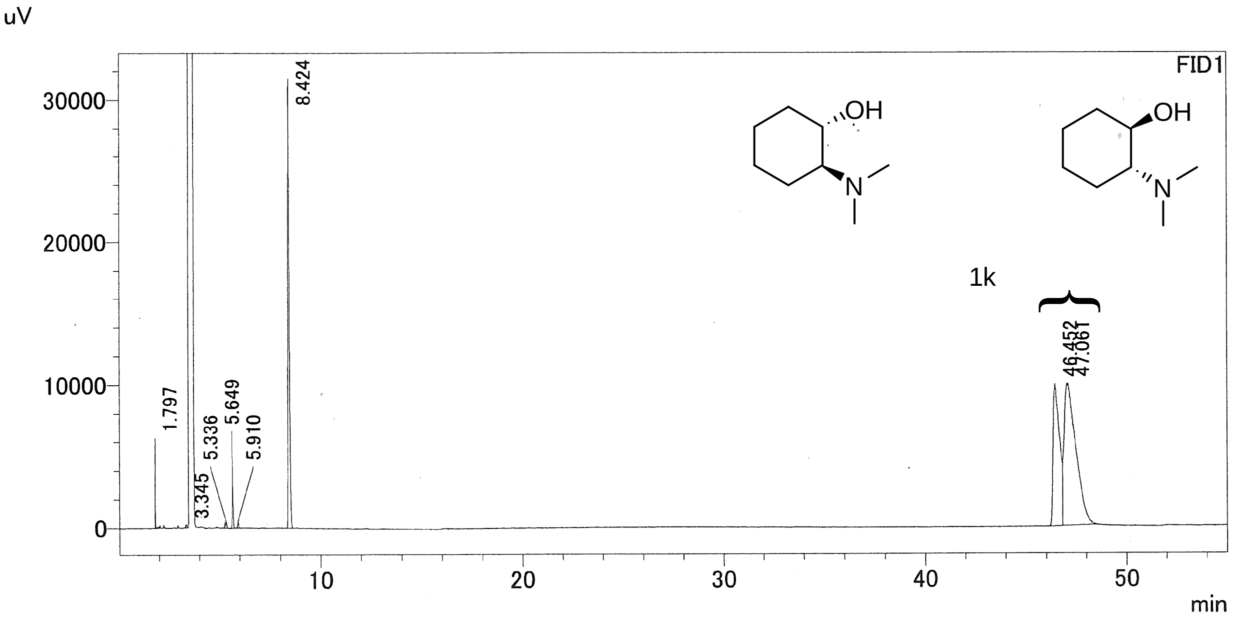

Peak Report

FID1

Peak#	Retention time	Area	Height	Area%	unit	Mark	Name
1	1.797	8873	4551	1.095			
2	3.345	1006	197	0.124			
3	5.336	2489	409	0.307			
4	5.649	9492	2667	1.172			
5	5.910	1497	396	0.185			
6	8.424	157398	30987	19.428			
7	46.452	222943	9883	27.518			
8	47.061	406464	9935	50.171		V	
Total		810162	59025				

Chiral GC chart of TMS-1m

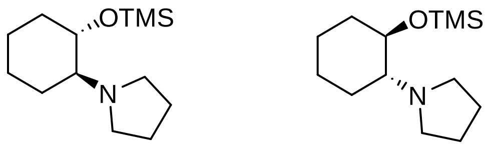

Chromatogram

TMS-1m

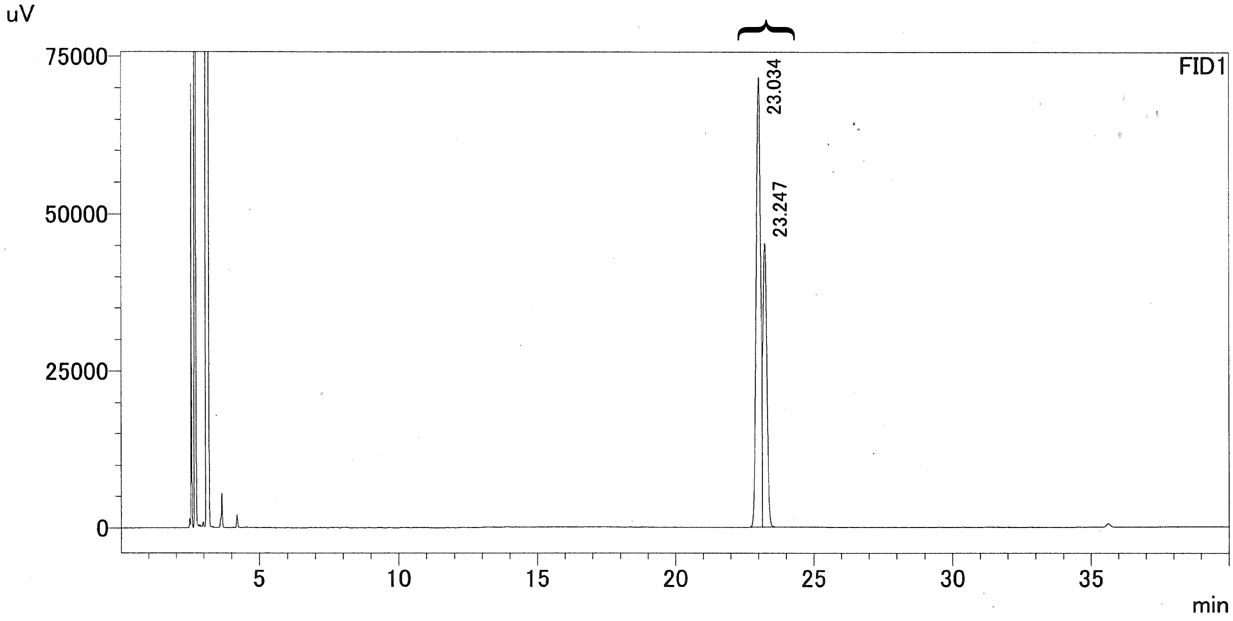

Peak Report

FID1

Peak#	Retention time	Area	Height	Area%	unit	Mark	Name
1	23.034	791790	71521	64.693			
2	23.247	432135	45171	35.307		V	
Total		1223925	116692				

Chiral HPLC chart of 1n

Chromatogram

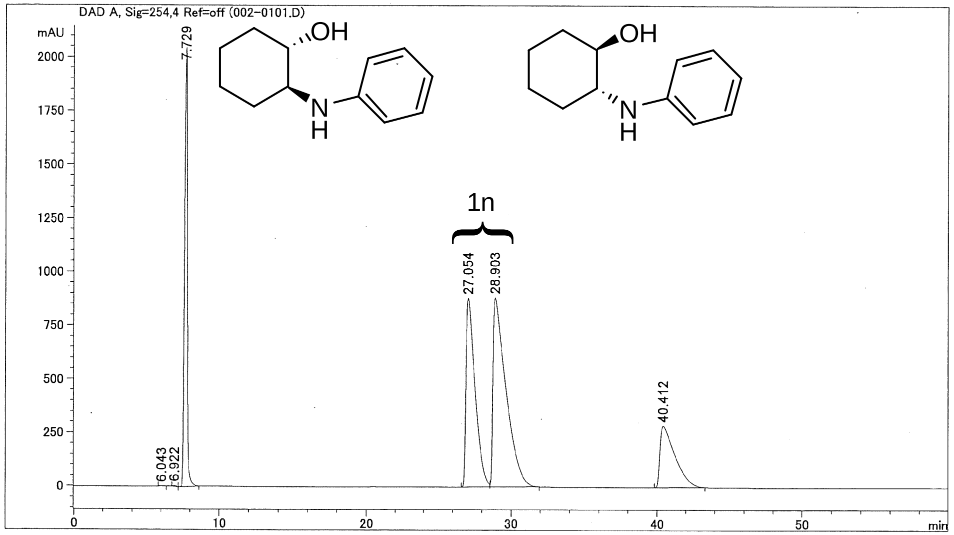

Peak Report

Signal 1: DAD B, Sig=254, 4 Ref=off

Peak #	RT [min]	Type	Width [min]	Area [mAU*s]	Height [mAU]	Area %
1	6.043	BB	0.1556	15.49496	1.35352	0.0110
2	6.922	BV	0.2062	83.24216	5.91270	0.0592
3	7.729	VB	0.2149	2.68113e4	2045.60474	19.0828
4	27.054	BV	0.6308	3.79297e4	880.14191	26.9962
5	28.903	VB	0.8483	5.50588e4	881.93048	39.1877
6	40.412	BB	1.0019	2.06015e4	285.48972	14.6630
Total :				1.40500e5	4100.43305	

Chiral GC chart of 1o

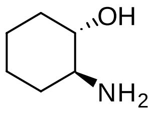

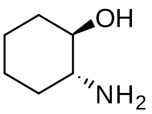

Chromatogram

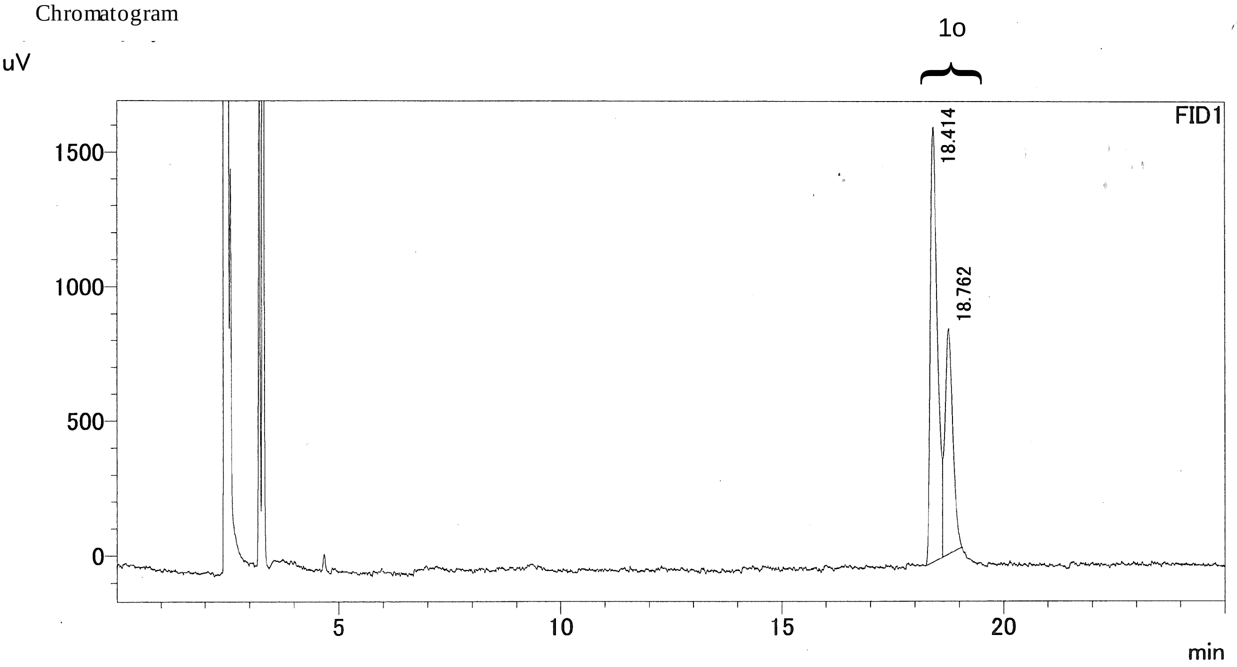

Peak Report

FID1

Peak#	Retention time	Area	Height	Area%	unit	Mark	Name
1	18.414	17782	1620	62.840			
2	18.762	10515	842	37.160		V	
Total		28298	2462				
